# Autistic People’s Perinatal Experiences II: A Survey of Childbirth and Postnatal Experiences

**DOI:** 10.1007/s10803-022-05484-4

**Published:** 2022-04-20

**Authors:** S. Hampton, C. Allison, S. Baron-Cohen, R. Holt

**Affiliations:** Department of Psychiatry, Autism Research Centre, University of Cambridge, Cambridge, UK

**Keywords:** Autism, Childbirth, Postnatal, Healthcare, Motherhood

## Abstract

Qualitative accounts indicate there are sensory and communication related barriers to adequate childbirth and postnatal healthcare for autistic people. However, little quantitative work has explored the topic. This online survey study explored childbirth and postnatal experiences among 384 autistic and 492 non-autistic people. Compared with non-autistic people, autistic people were more likely to find the sensory aspects of birth overwhelming, and experienced lower satisfaction with birth-related and postnatal healthcare. Autistic people were more likely to experience postnatal depression and anxiety. The findings highlight that sensory and communication adjustments should be made to birth and postnatal healthcare for autistic people. The findings indicate the need for greater autism understanding among professionals and greater postnatal mental health support for autistic people.

**Supplementary Information:**

The online version contains supplementary material available at 10.1007/s10803-022-05484-4.

## Background

There has been very little work exploring autistic people’s childbirth and postnatal experiences. The experiences of those with disabilities, including those with mental health conditions and those with intellectual disability, are better documented and may help to inform understanding of autistic people’s experiences, given that such conditions often co-occur with autism and may bring similar challenges (Lai et al., 2019; Rydzewska et al., 2019).

Those with disabilities have been found to be at increased risk of poorer birth outcomes. For example, those with physical disabilities are more likely to have assisted vaginal births, planned caesarean sections and emergency caesarean sections compared with those without disabilities (Malouf et al., 2017). Furthermore, people with intellectual disability are more likely to have caesarean or induced births than people without intellectual disability (Brown et al., 2016) and to have poorer birth outcomes such as preterm delivery and low birth weight (Mitra et al., 2015). One study has explored birth outcomes for autistic people. Using Swedish national medical data from 2006–2014, this study compared the birth outcomes of 2198 autistic people and 877,742 non-autistic people (Sundelin et al., 2018). They found that autistic people had increased risk of moderately preterm birth (32 to < 37 weeks), but no difference in risk of preterm birth from 28 to < 32 weeks or preterm birth below 28 weeks. This increased risk in moderately preterm birth was likely due to increased risk of medically indicated preterm birth (preterm birth due to induction of labour or caesarean section before labour); no risk of increased spontaneous preterm birth was found. Autistic people were more likely to have an elective caesarean and more likely to have induced labour than non-autistic people.

Recent studies have identified gaps in disabled people’s childbirth and postnatal healthcare. For example, based on UK survey data from 2015, one paper found that disabled people (including those with physical disabilities, mental health conditions, sensory disabilities and intellectual disabilities) had less favourable perceptions of birth care (Malouf et al., 2017). In particular, people with physical, mental health and learning disabilities were less likely to have trust in staff, less likely to be spoken to by staff in a way they could understand, and less likely to report always being treated with respect by staff. Those with mental health and intellectual disabilities were also less likely to report that their concerns were taken seriously by staff during labour and birth. Low perceptions of healthcare were also common postnatally. During postnatal appointments, those with disabilities were less likely to feel listened to by professionals, to have trust in their midwives, and to receive the help they needed from midwives. Those with physical and mental health disabilities were also less likely to report being treated with kindness and understanding during their postnatal hospital care, less likely to report receiving support for infant feeding during their hospital stay and during the six weeks after birth, and less likely to have received sufficient information about their physical recovery after birth or possible mood changes after birth.

An analysis of UK national survey data from 2010, also found less favourable perceptions of perinatal care among people with disabilities, particularly those with mental health and learning disabilities (Redshaw et al., 2013). In addition, this study further indicated that disabled people were less likely than non-disabled people to breastfeed and less likely to be given the pain relief they wanted during labour. A further survey study focusing on those with mental health conditions found that they had lower satisfaction concerning the experience of birth and perceived maternity care less positively than those without mental health conditions (Henderson et al., 2018). This included being less likely to feel that doctors talked to them in a way they could understand, treated them respectfully and listened to them.

Furthermore, research exploring healthcare professionals’ perspectives has revealed that midwives do not feel they have sufficient training to provide appropriate care for those with mental health conditions (Noonan et al., 2018) nor intellectual disability (Homeyard et al., 2016). In addition, there is evidence from Swedish data that midwives can possess negative attitudes such as the belief that people with intellectual disability cannot satisfactorily manage the role of being a mother (Höglund et al., 2013).

Studies exploring access to healthcare for autistic people have identified sensory-related barriers to healthcare (such as difficulties with the sensory environment of healthcare facilities (Raymaker et al., 2017)), as well as communication barriers such as difficulty processing verbal information (Raymaker et al., 2017) and a need for more accessible communication formats such as written information (Nicolaidis et al., 2015). In addition, professionals across a variety of areas of healthcare report that they lack adequate knowledge and training about autism in adults (Morris et al., 2019; Urbanowicz et al., 2020). Studies focusing specifically on autistic experiences of maternity care have tended to echo these findings of sensory and communication barriers to appropriate healthcare. For example, a case study of one Australian autistic woman’s experiences reported that the woman found it challenging to cope with being touched by professionals during the birth (Rogers et al., 2017). She also reported difficulties communicating with health professionals during postnatal appointments, who she felt did not take her concerns seriously, did not treat her respectfully, and judged her parenting ability negatively due to being autistic.

Another study retrospectively explored the perinatal experiences of eight autistic women (Gardner et al., 2016). The mothers reported that, during childbirth, they experienced sensory difficulties with bright lights and the sounds of other women in labour. Sensory issues surrounding touch were also identified as making breastfeeding challenging. The mothers reported that they did not always disclose their autism diagnosis to professionals and that they required direct and clear information when interacting with professionals. They described not having had sufficient support for caring for their infant, such as understanding their baby’s facial expressions and connecting emotionally with their baby. They also felt that others had judged their parenting and desired to approach parenting on their ‘own terms’ rather than following others’ expectations. A study of 7 autistic mothers also reported sensory challenges during childbirth, such as difficulties with being touched and bright lights, and that the autistic mothers interviewed often did not feel that medical professionals understood or accommodated their sensory needs (Talcer et al., 2021). A further study reported on interviews with 24 autistic women from the USA, UK and Australia who had given birth within the previous 10 years about their experience of childbirth (Donovan, 2020). Participants expressed difficulty communicating with professionals, including difficulty conveying needs and understanding what was said to them. Difficulties in communication often led to feelings of anxiety and inhibited future attempts at communication.

Quantitative literature exploring autistic mothers’ experiences is scarce. One survey study found that autistic mothers experienced communication difficulties with professionals (e.g. teachers, clinicians, social workers) (Pohl et al., 2020). They were also more likely to feel misunderstood by professionals and were reluctant to disclose their autism diagnosis for fear that professionals would change their attitude towards them if they did so. Autistic mothers were also more likely to experience postnatal depression and less likely to feel that the process of birth was adequately explained to them. Autistic mothers were just as likely to attempt to breastfeed, though were more likely to have difficulties breastfeeding their second child.

There is currently no in-depth quantitative research focusing on the childbirth and postnatal experiences of autistic people. This study aimed to explore perceptions of birth and postnatal healthcare among autistic people, in order to identify gaps in current practice. The survey also aimed to explore birth outcomes and autistic people’s physical and sensory experiences during childbirth.

## Methods

### The survey

The survey contained three sections: pregnancy, childbirth and postnatal experiences. This paper reports on the childbirth and postnatal sections, while the pregnancy section is reported on elsewhere (Hampton et al., under review). The childbirth section covered: (1) birth outcomes; (2) sensory and physical aspects of birth; (3) healthcare experiences and (4) postnatal hospital stay. The postnatal section covered: (1) postnatal health; (2) breastfeeding; (3) postnatal appointments; (4) support.

The survey contained forced choice and open-ended questions. The forced choice questions most often required one of: ‘strongly agree’, ‘somewhat agree’, ‘somewhat disagree’, ‘strongly disagree’, ‘don’t know’ or ‘not applicable’. Some questions were presented depending on the response given to a previous question. For example, ‘I have had difficulties breastfeeding my baby’ was only asked if respondents indicated they had breastfed. Questions concerning autism were only asked to those in the autistic group. The survey also contained demographic questions and the 10-item version of the Autism Quotient (AQ-10 (Allison et al., 2012)), a self-report measure of autistic traits. Scores on the AQ-10 range from 0 to 10, with a score of six or above indicating that a clinical assessment for autism may be warranted.

The findings from a separate qualitative study exploring autistic women’s perinatal experiences (Hampton et al., 2022) were used as a foundation for choosing the topics covered. Additionally, feedback from the autistic community was sought through Twitter. Comments on which aspects of pregnancy autistic followers would like to see more research on were taken into account when creating the survey. Three autistic mothers gave feedback on the survey. Each of the mothers worked with other autistic mothers in a professional capacity, one as a midwife, another as a doula and another as a researcher. Feedback was gained through email exchanges concerning the phrasing and content of the questions. A final draft of the survey was also piloted with five non-autistic mothers to help ensure that the content was appropriate and that the language used was clear. These mothers expressed overall satisfaction with the survey and minor changes were made based on their feedback. These changes included altering ambiguous language and including questions about postnatal symptoms.

Participants completed the survey online and indicated their informed consent electronically. Responses were anonymous and ethical approval was obtained from the University of Cambridge Ethics Committee, PRE.2018.093.

### Participants

Participants were recruited through the Cambridge Autism Research Database (CARD), parenting groups, autism support groups and social media. Participants were eligible to complete the childbirth questions if they were at least 18 years old and had given birth at least once. Participants were requested to reflect on their most recent birth experience. Respondents were asked to fill in the postnatal questions if they had a child who was at least three months old at the time of completing the survey. Participants were asked to reflect on their experience with their youngest child who they gave birth to.

In total, 231 people with a diagnosis of autism, 153 people who believed themselves to be autistic but did not have a diagnosis and 490 non-autistic people (who neither had a diagnosis nor believed themselves to be autistic) were included in the study. Post-hoc sensitivity power analysis indicated that for the total sample (n = 874), there was adequate (80%) power to detect small effect sizes (odds ratio ≥ 1.52), with a two-tailed alpha of 0.05.

Those who believed themselves to be autistic but did not have a diagnosis were included in the autistic group. This is because the mean AQ-10 score of the self-identifying group was above the cut-off of six (mean = 7.01, SD = 2.11) and, even though their AQ-10 mean score was significantly lower than that of those with a diagnosis (mean = 7.91, SD = 1.66, *p* < 0.001), they scored significantly higher than the non-autistic group (mean = 1.95, SD = 1.66, *p* < 0.001). This approach follows that of a previous similar paper (Pohl et al., 2020). Descriptive statistics are presented separately for participants with an autism diagnosis and participants who self-identify as autistic in Online Resource 1.

The autistic and non-autistic groups did not differ significantly on current age, education, ethnicity, whether their most recent pregnancy was singleton or multiple, total number of pregnancies or total number of live births (Table 1). The groups significantly differed on age at most recent birth, current partner status and country of residence. The autistic group were significantly more likely to identify as non-binary/other gender, had significantly lower annual household income, were significantly more likely to have been diagnosed with a psychiatric condition and gave birth to their youngest child significantly longer ago than the non-autistic group.

**Table 1:** Demographic information for the autistic and non-autistic groups

	Non-autistic group	Autistic group	*p* value
Mother’s current age^a^			0.15
N	490	384	
Mean (SD)	41.54 (9.88)	42.46 (9.14)	
Mother’s age at most recent birth^a^			**0.002**
N	490	384	
Mean (SD)	33.05 (5.03)	31.95 (5.32)	
Gender identity^b^			** < 0.001**
N	490	384	
Female	488 (100%)	358 (93%)	
Male	1 (0.20%)	1 (0.26%)	
Non-binary/other	1 (0.20%)	25 (7%)	
Education^b^			0.23
N	490	384	
Completed high school	88 (18%)	79 (21%)	
Undergraduate degree	202 (41%)	142 (37%)	
Postgraduate degree	178 (36%)	136 (35%)	
Other	22 (5%)	27 (7%)	
Income^b^			** < 0.001**
N	490	384	
Greater than £100,000	84 (16%)	40 (10%)	
£50,000-£100,000	179 (37%)	92 (24%)	
£25,000-£50,000	150 (31%)	129 (34%)	
Less than £25,000	77 (17%)	123 (32%)	
Current partner status^b^			** < 0.001**
N	490	384	
Married/in a partnership	429 (88%)	291 (76%)	
Divorced/separated/widowed	34 (7%)	55 (14%)	
Single	27 (6%)	38 (10%)	
Country^b^			** < 0.001**
N	490	384	
UK	349 (71%)	230 (60%)	
USA	48 (10%)	80 (21%)	
Ireland	51 (10%)	11 (3%)	
Other	42 (9%)	63 (16%)	
Ethnicity^b^			0.93
N	487	381	
White	461 (95%)	361 (95%)	
Non-white	26 (5%)	20 (5%)	
* Asian*	7 (1%)	1 (0.26%)	
* Black African/Black* * Caribbean*	1 (0.20%)	0 (0%)	
* Mixed ethnicity*	12 (2%)	8 (2%)	
* Other*	6 (1%)	11 (3%)	
Psychiatric condition(s)^b^			** < 0.001**
N	490	384	
Yes	184 (38%)	259 (67%)	
No	306 (62%)	125 (33%)	
AQ-10 score^c^			** < 0.001**
N	490	384	
Mean (SD)	1.95 (1.66)	7.55 (1.91)	
Total number of pregnancies^c^			0.62
N	490	384	
Mean (SD)	2.93 (1.79)	3.08 (2.02)	
Total number of live births^c^			0.31
N	490	384	
Mean (SD)	1.99 (1.07)	2.10 (1.20)	
Age of youngest child in years^c^			** < 0.001**
N	490	384	
Mean (SD)	8.39 (8.36)	10.48 (8.62)	
Singleton or multiple birth (youngest child)^b^			0.64
N	490	384	
Singleton	478 (98%)	377 (98%)	
Multiple	12 (2%)	7 (2%)	

*SD* standard deviation

^a^T-test performed

^b^Fisher’s exact test performed

^c^Wilcoxon rank-sum test performed

*p*-values in bold are significant at *p* < 0.05

### Data analysis

Ineligible participants were excluded, including those under 18 years old (1 participant) and those who had never given birth (13 participants). Participants were excluded if they were suspected to be duplicates, that is, if they had the same identifying code as another participant and gave the same demographic responses (30 participants). Duplicates may have arisen due to participants re-starting the survey after the initial link expired. Anyone who had not answered at least 20 percent of the survey questions beyond the demographic questions was excluded (197 participants).

‘Strongly agree’ and ‘somewhat agree’ responses were combined into an ‘agree’ category and ‘strongly disagree’ and ‘somewhat disagree’ were combined into a ‘disagree’ category, in order to facilitate analysis with logistic regression, and for ease of interpretation of results. Similarly, ‘very satisfied’ and ‘somewhat satisfied’ were reduced to ‘satisfied’, and ‘very dissatisfied’ and ‘somewhat dissatisfied’ were reduced to ‘dissatisfied’. This approach of collapsing Likert scales to two categories and performing binary logistic regression has been taken in prior survey studies of perinatal experiences (e.g. Redshaw et al., 2013). ‘Don’t know’ and ‘Not applicable’ responses were excluded from analysis.

Where possible, thematically similar items were analysed in a multivariate manner in order to account for correlations among items. This was achieved by reshaping the data into long format such that responses for all items were aggregated into one binary (agree/disagree) outcome variable. In this manner, items were effectively treated as repeated measures. A multilevel binary logistic regression was then performed with the agree/disagree response variable as the outcome and group as a predictor. Each model included a random intercept for participant to account for dependency due to repeated measures. A group by item interaction term was included in each model in order to obtain odds ratios and confidence intervals for each individual item. Items that correlated negatively with the other items within the multivariate analysis were reverse scored prior to analysis. To obtain an omnibus analysis of the effect of group across the items as a whole, a likelihood ratio test was performed comparing the model with group as a predictor and the model without group as a predictor; if the model with group as a predictor was a significantly better model than that without, group was considered to have a significant effect on responses across the items as a whole. Only if this omnibus test was significant were analyses relating to individual items presented.

Decisions to group items together in a multivariate analysis were based on thematic similarity between items (e.g. questions regarding childbirth experiences were analysed together, questions regarding breastfeeding were analysed together etc.). Polychoric correlations between theoretically related items were also conducted (see Online Resource 2). Thematically similar items were generally at least moderately correlated (r ≥ 0.30 was considered moderate (Cohen, 1992)), supporting a multivariate analysis. Some items were only weakly correlated with others and were excluded from the multivariate analysis. For example, items concerning attending appointments were strongly correlated with each other though had few correlations of r ≥ 0.30 with other postnatal healthcare items, and therefore were analysed together in a multivariate analysis but excluded from the main postnatal healthcare multivariate analysis.

Some items that were survey logic dependent (only presented depending on the response to a prior question) were excluded from multivariate analyses. For example, the question, ‘I found it helpful to have access to sensory items’ was asked only if participants previously indicated having access to sensory items during birth and the question, ‘I would have found it helpful to have access to sensory items’ was asked if participants indicated not having access. These questions were therefore not entered together into a multivariate analysis and were analysed individually.

Correction for multiple comparisons was not applied to analyses of individual items within a multivariate analysis, though all other analyses were FDR corrected (i.e. all analyses of individual items not included within a multivariate analysis and omnibus tests of the overall effect on group on multiple items were corrected for together). Questions within the birth section and questions within the postnatal section were corrected for separately. *p* values of less than 0.05 are considered significant.

All analyses included the following covariates: mothers’ age at giving birth, time passed since giving birth (age in days of their youngest biological child), the number of live births the participant had experienced, country of residence, income, current partner status and the presence of one or more psychiatric conditions (yes or no). For questions concerning birth experiences, gestational age at birth and type of delivery (vaginal, assisted vaginal, elective caesarean or emergency caesarean) were included as covariates. Correlations between covariates were conducted and none were highly correlated (all were r = 0.30 or below). The adjusted odds ratio (aOR) is reported for each analysis. Those participants with missing data for any covariate were excluded from analyses (25 participants for the postnatal questions and an additional 21 participants for the birth questions).

While the quantitative data are the focus of this paper, quotes from the open-text responses are also reported in order to elucidate the quantitative data. A full qualitative analysis (Braun & Clarke, 2006) was not conducted and as such, the open-text response data are intended to provide preliminary, speculative elucidation of the quantitative findings.

## Results

### Childbirth Experiences

#### Birth Outcomes: Delivery Type and Gestational Age

There were no significant group differences in delivery type nor gestational age at birth (Table 2).

**Table 2:** Delivery type and gestational age

	Non-autistic group (n = 480)	Autistic group (n = 373)	aOR (95% CI)	*p* value	*p* value (FDR adjusted)
Delivery type:^a^					
Vaginal	301 (63%)	242 (65%)	1.04 (0.76–1.45)	0.79	0.92
Assisted vaginal	51 (11%)	34 (9%)	1.02 (0.60–1.70)	0.95	0.95
Elective caesarean	49 (10%)	38 (10%)	1.06 (0.64–1.74)	0.82	0.92
Emergency caesarean	76 (16%)	57 (15%)	0.96 (0.63–1.47)	0.86	0.92
Induced	108 (23%)	92 (25%)	1.11 (0.77–1.58)	0.58	0.87
	Non-autistic group (n = 480)	Autistic group (n = 373)	B (SE)	*p* value	*p* value (FDR adjusted)
Mean gestational age at birth (days)(SD)^b^	276 (17.80)	275 (15.20)	1.01 (1.23)	0.41	0.68

*aOR* adjusted odds ratio, *CI* confidence intervals, *FDR* false discovery rate, *B* beta, *SE* standard error

^a^Binary logistic regressions performed

^b^Multiple linear regression performed

#### Childbirth Experiences

For questions concerning birth experiences, a multivariate binary logistic regression was performed. A model including group as a covariate was a better fit than the model without group, *X*^2^(9) = 206.16, *p* < 0.001, indicating that the groups significantly differed.

The autistic group were significantly more likely to feel overwhelmed by sensory input during birth (65% vs. 29%; Online Resource 3, Supplementary Table 1). There was no significant group difference in having access to sensory items (such as a weighted blanket, scented oil, fidget toys etc.). However, for those who did not have access to sensory items, the autistic group were significantly more likely to feel that these items would have been helpful (50% vs. 17%). The autistic group were significantly less likely to agree that they felt aware of their body’s signals and how to correctly interpret them during birth (52% vs. 65%).

The autistic group were also significantly more likely than the non-autistic group to have experienced a meltdown (29% vs. 17%) and a shutdown (38% vs. 8%). A meltdown can be defined as becoming overwhelmed by the current situation and expressing this verbally (e.g. shouting, screaming, crying) or physically (e.g. kicking, lashing out, biting). Shutdowns can be defined as becoming overwhelmed and withdrawing from the world around oneself, for example being unable to communicate, lying down and being completely still and not being able to move. However, there were no significant group differences in the likelihood of agreeing that professionals responded to their meltdown (33% vs. 59%) or shutdown (35% vs. 50%) how they would have liked. When asked to describe how they would have liked medical professionals to respond, the autistic group particularly highlighted a need for more understanding of shutdowns, ‘*When I was crying/shouting they seemed to understand what I was feeling, but most of the time I was shut down and silent and they didn't seem to understand that it was a shutdown and that I wasn't able to focus on anything in the room or understand anything being asked of me’*.

Regarding relationships with professionals, the autistic group were significantly less likely to agree that they were kept adequately informed by professionals (55% vs. 73%), that professionals listened to their requests (57% vs. 75%), that professionals had an accurate understanding of what they were perceiving physically (40% vs. 72%) and they were more likely to agree that they felt pressure to behave in a socially normative way during the birth (64% vs. 34%).

The groups did not significantly differ on whether or not they made a birth plan, though the autistic group were significantly less likely to agree that medical professionals took their birth plan into account (52% vs. 65%). The groups did not differ on whether or not they had someone to advocate for them (for example, a partner, friend or family member) during the birth (71% of the autistic group and 75% of the non-autistic group did). For those who had an advocate, the groups did not differ in their tendency to agree that having an advocate was helpful (82% of the autistic group and 87% of the non-autistic group agreed). For those who did not have an advocate, the autistic group were significantly more likely to agree that having an advocate would have been helpful (64% vs. 33%). The autistic group were also significantly less likely to feel satisfied with the medical care they received during childbirth (71% vs. 86%).

When asked whether professionals had a good understanding of how being autistic affected them during the birth, the majority (65%) of autistic participants felt that this questions was not applicable to them (possibly due to not being diagnosed at the time of their most recent birth). 20% disagreed and 2% agreed that professionals had a good understanding of how autism affected them.

#### Postnatal Hospital Stay

For questions concerning postnatal hospital experiences, a multivariate binary logistic regression was performed. A model including group as a predictor was a better fit than the model without group, *X*^2^(2) = 61.68, *p* < 0.001. Of those who indicated that they stayed on a shared postnatal ward at the hospital, the autistic group were significantly more likely to agree that they found this overwhelming in terms of sensory input (88% vs. 61%; Table 3). The autistic group were significantly less likely to feel satisfied with the services they received during their postnatal stay (53% vs. 69%).

**Table 3:** Postnatal hospital stay

	Non-autistic group	Autistic group	aOR (95% CI)	*p* value
I found being on a shared postnatal ward overwhelming in terms of sensory input^a^			7.41 (3.86–14.27)	** < 0.001**
N	274	186		
Agree	168 (61%)	163 (88%)		
Disagree	99 (36%)	21 (11%)		
Don’t know	2 (1%)	0 (0%)		
Not applicable	5 (2%)	2 (1%)		
Overall, how satisfied were you with the services you received during your postnatal stay?			0.32 (0.21–0.49)	** < 0.001**
N	464	358		
Satisfied	318 (69%)	190 (53%)		
Dissatisfied	103 (22%)	130 (36%)		
Don’t know	0 (0%)	4 (1%)		
Not applicable	43 (9%)	34 (10%)		

*Note.* Multivariate binary logistic regression performed

*p*-values in bold are significant at *p* < 0.05

^a^Item reverse scored prior to multivariate analysis. Inverse of aOR and CIs presented

### Postnatal Experiences

#### Postnatal Physical and Mental Health

Questions about postnatal physical symptoms were explored with multivariate binary logistic regression. A model including group as a covariate was a better fit than the model without group, *X*^2^(2) = 57.23, *p* < 0.001. The autistic group were significantly less likely to feel prepared to cope with physical postnatal symptoms (56% vs. 73%; Table 4). The autistic group were also significantly less likely to have known when to seek help with postnatal symptoms (60% vs. 83%). The autistic group were significantly more likely to have been told by a professional that they had postnatal depression (30% vs. 13%) and postnatal anxiety (19% vs. 7%).

**Table 4:** Postnatal physical and mental health

	Non-autistic group	Autistic group	aOR (95% CI)	*p* value	*p* value (FDR adjusted)
I felt prepared to cope with physical postnatal symptoms after giving birth			0.26 (0.15–0.45)	** < 0.001**	–
N	435	357			
Agree	316 (73%)	201 (56%)			
Disagree	115 (26%)	152 (43%)			
Don’t know	0 (0%)	2 (1%)			
Not applicable	4 (1%)	2 (1%)			
I have known when to seek help with physical postnatal symptoms			0.12 (0.07–0.23)	** < 0.001**	–
N	435	357			
Agree	360 (83%)	215 (60%)			
Disagree	65 (15%)	132 (37%)			
Don’t know	2 (0.46%)	7 (2%)			
Not applicable	8 (2%)	3 (1%)			
Were you told by a medical/health professional that you had postnatal depression?^a^			2.31 (1.54–3.49)	** < 0.001**	** < 0.001**
N	435	355			
Yes	55 (13%)	108 (30%)			
No	380 (87%)	247 (70%)			
Were you told by a medical/health professional that you had postnatal anxiety?^a^			1.92 (1.16–3.24)	**0.01**	**0.02**
N	434	356			
Yes	32 (7%)	69 (19%)			
No	402 (93%)	287 (81%)			

*Note.* Multivariate binary logistic regression performed

*p*-values in bold are significant at *p* < 0.05

^a^Item not included within multivariate analysis

#### Breastfeeding Experiences

For questions concerning breastfeeding, a multivariate binary logistic regression was performed. A model including group as a predictor was a better fit than the model without group, *X*^2^(3) = 21.33, *p* < 0.001. The autistic group were more likely to have breastfed or attempted to breastfeed (94% vs. 91%; Online Resource 3, Supplementary Table 2). Among those who had breastfed, the groups did not significantly differ on having had difficulties breastfeeding (60% of the autistic group and 57% of the non-autistic group). Among those who had experienced difficulties breastfeeding, the autistic group were significantly more likely to have had difficulties due to sensory issues (47% vs. 10%).

Among those who breastfed, the autistic group were significantly less likely to agree that they found it easy to access support (48% vs. 60%) and were significantly less likely to be satisfied with the support they received (48% vs. 56%).

#### Postnatal Appointments

##### Autism Disclosure, Adjustments and Autism Understanding

When asked whether they had disclosed their autism during postnatal appointments, the majority of autistic participants considered the question not applicable (Table 5). Of those who felt the question was applicable, the majority had not disclosed. 26% did not disclose to their midwife (7% disclosed), 26% did not disclose to their health visitor (8% disclosed) and 27% did not disclose to their doctor/GP (11% disclosed). When asked what had influenced their decision, in their open-text responses many commented that they were not diagnosed at the time. Those who did not disclose sometimes described fear that disclosure would lead to discrimination, ‘*I would never disclose for fear of discrimination. I’ll tell a perfect stranger before I’ll tell a doctor or a nurse, because I want to be believed*’.

**Table 5:** Autism disclosure, adjustments and autism understanding at postnatal appointments

	N	Yes	No	Not applicable	
Disclosed autism to:					
Midwife	350	25 (7%)	90 (26%)	235 (67%)	
Health visitor	350	29 (8%)	91 (26%)	230 (66%)	
Doctor/GP	352	40 (11%)	94 (27%)	218 (62%)	
Adjustments offered	50	17 (34%)	33 (66%)	–	
Adjustments desired that were not offered	50	23 (46%)	27 (54%)	–	
	N	Agree	Disagree	Don’t know	Not applicable
Health professionals have had a good understanding of how being autistic affects me:					
Midwife	348	25 (7%)	32 (9%)	38 (11%)	253 (73%)
Health visitor	346	22 (6%)	38 (11%)	41 (12%)	245 (71%)
Doctor/GP	349	31 (9%)	49 (14%)	46 (13%)	223 (64%)

Of those who had disclosed, 34% indicated that adjustments were made for them. Participants indicated in their open-text responses that these adjustments included home visits, longer appointments, accommodating sensory issues and giving information in a visual format. Among those who had disclosed, 46% agreed that there were adjustments they would have liked but that were not offered to them. When asked to give an open-text response what adjustments would have been helpful, participants mentioned home visits, longer appointments, dimming the lights in appointments, giving written information, and being able to book appointments through another method than telephone.

When asked whether health professionals had a good understanding of how being autistic affects them, most participants indicated that the question was not applicable (possibly due to not being diagnosed). A small minority of autistic participants agreed that their midwife, health visitor or doctor/GP had a good understanding of autism (7%, 6% and 9% respectively).

##### Attending Postnatal Appointments

For questions concerning attending postnatal appointments, a multivariate binary logistic regression was performed. A model including group as a covariate was a better fit than the model without group, *X*^2^(4) = 14.21, *p* = 0.01. However, individual analyses of each appointment type revealed no significant group differences (Table 6).

**Table 6:** Attending postnatal appointments

	Non-autistic group	Autistic group	aOR (95% CI)	*p* value
Attended all				
midwife appointments			7.69 (0.65–90.70)	0.11
N	433	352		
Yes	367 (85%)	274 (78%)		
No	14 (3%)	9 (3%)		
Not applicable	52 (12%)	69 (20%)		
Attended all health visitor appointments			0.32 (0.08–1.80)	0.20
N	432	350		
Yes	387 (90%)	272 (78%)		
No	15 (3%)	20 (6%)		
Not applicable	30 (7%)	58 (17%)		
Attended mother’s 6 week check			11.36 (0.95–194.00)	0.06
N	432	352		
Yes	407 (94%)	329 (94%)		
No	11 (3%)	8 (2%)		
Not applicable	13 (3%)	15 (4%)		
Attended baby’s 6–8 week check			7.39 (0.36–153.00)	0.20
N	428	353		
Yes	417 (97%)	341 (97%)		
No	4 (1%)	5 (1%)		
Not applicable	7 (2%)	7 (2%)		

*Note*. Multivariate binary logistic regression performed

##### Other Aspects of Postnatal Appointments

The remaining questions concerning postnatal appointments were explored with a multivariate binary logistic regression. A model including group as a predictor was a better fit than the model without group, *X*^2^(19) = 330.66, *p* < 0.001.

The autistic group were significantly more likely to indicate that they found it stressful to have professionals visit their home, such as midwives and health visitors who complete postnatal checks (63% vs. 22%; Online Resource 3, Supplementary Table 3). Both groups were just as likely to see the same professional at each postnatal appointment e.g. the same midwife at each midwife appointment (39% vs. 40%), though the autistic group were significantly more likely to agree that seeing the same professional at each appointment was important to them (89% vs. 76%). The autistic group were also significantly more likely to have found it stressful when the professional they saw was not who they were expecting to see (59% vs. 31%).

The autistic group were significantly less likely to agree that professionals took their questions and concerns seriously (59% vs. 82%), to have felt comfortable asking questions (58% vs. 85%), to feel that professionals treated them respectfully (71% vs. 90%) and to have felt able to trust professionals (56% vs. 82%). 49% of the autistic group felt negatively judged by professionals during postnatal appointments, significantly more than the non-autistic group (23%).

The autistic group were significantly less likely to have received as much information as they would have liked during postnatal appointments about their mental health (36% vs. 60%), looking after a baby (30% vs. 43%), how to interpret a baby’s cries (59% vs. 70%) and how to play with a baby (34% vs. 44%). The autistic group were also significantly less likely to be satisfied with the way in which information was presented (58% vs. 80%).

The groups did not significantly differ on whether or not they had someone to advocate for them at postnatal appointments. Among those who had an advocate, the groups did not differ on whether they agreed that this was helpful (85% of the autistic group and 79% of the non-autistic group agreed). However, among those who did not have an advocate, the autistic group were significantly more likely to agree that an advocate would have been helpful (57% vs. 23%).

The autistic group were less likely to be satisfied with their midwife appointments (60% vs. 78%), health visitor appointments (51% vs. 72%) and doctor/GP appointments (62% vs. 81%). The autistic group were significantly more likely to have found it difficult to attend drop-in clinics to get their baby weighed (49% vs. 29%) and parent and baby groups (80% vs. 41%).

##### Impact of Disclosure on Postnatal Healthcare

Results of analyses exploring the impact of disclosure of an autism diagnosis on postnatal healthcare are presented in Online Resource 4. A multivariate binary logistic regression revealed that whether or not participants disclosed their diagnosis did not significantly predict responses to the postnatal appointments questions as a whole.

### Postnatal Support

For questions concerning postnatal support, a multivariate binary logistic regression was performed. A model including group as a covariate was a better fit than the model without group, *X*^2^(3) = 41.05, *p* < 0.001. The autistic group were less likely to feel they had all the support they needed from their partner/spouse (52% vs. 72%), family (44% vs. 71%) and friends (42% vs. 71%; Online Resource 3, Supplementary Table 4). The majority (83%) of autistic participants did not have peer support from other autistic parents, though 98% of those who did agreed that they had found it helpful and 60% of those who did not have peer support agreed that they would have found such support helpful.

## Discussion

This is the first quantitative study focusing on the childbirth and postnatal experiences of autistic people. Evidence was found of atypical sensory and physical experiences during childbirth among autistic people in addition to less favourable perceptions of birth related and postnatal healthcare.

In terms of physical birth outcomes, no increased risk of induction or caesarean delivery was found in the present sample, in contrast with findings from Sundelin et al. (2018). Sundelin et al. (2018) suggest that increased risk of induction and elective caesarean may be due to a tendency for professionals to commence labour based on maternal wellbeing, possibly due to the stresses of different sensory processing among autistic people. If this is the case, this phenomenon may be less common in non-Swedish healthcare systems (such as the UK and other countries represented in the survey). It is also possible that the present study is underpowered to detect differences in birth outcomes. The groups did differ, however, on their reports of the physical experiences of birth. For example, in keeping with prior findings (Gardner et al., 2016), autistic people were more likely to feel overwhelmed by sensory input during childbirth. Similarly, autistic participants were more likely to feel overwhelmed by the sensory environment of a shared postnatal ward. These data highlight the need to make sensory accommodations for autistic people during childbirth and on the postnatal ward. The autistic group were also less likely to feel able to cope with physical symptoms following childbirth and less likely to know when to seek help with these. This complements prior findings that people with disabilities are less likely to receive sufficient information about physical recovery after birth (Malouf et al., 2017) and indicates that autistic people would benefit for greater information surrounding postnatal recovery.

The majority of autistic participants did not feel that professionals had an accurate understanding of what they were perceiving physically during birth and the autistic group were also less likely to feel aware of their bodily signals during labour. These findings suggest that professionals may need to communicate differently with autistic and non-autistic patients about their bodily signals during childbirth.

Autistic participants were less likely to feel that their birth plan was taken into account and their requests listened to during childbirth. This is in keeping with previous findings that those with disabilities are less likely to feel that their concerns are taken seriously during labour (Henderson et al., 2018; Malouf et al., 2017). Furthermore, autistic participants were less likely to feel kept informed during the birth. This echoes previous findings that autistic mothers are less likely to feel that the process of birth was adequately explained (Pohl et al., 2020) as well as findings that those with disabilities are less likely to be spoken to in a way they could understand during childbirth (Malouf et al., 2017). These results indicate the need for communication adjustments for autistic people during childbirth. These adjustments could include clearer explanations and more time to process information, given that autistic people can find processing verbal information challenging in healthcare contexts (Nicolaidis et al., 2015; Raymaker et al., 2017). It should be noted, however, that these accommodations may be less feasible during certain aspects of labour, such as the final stages.

Approximately a third of autistic participants reported having a meltdown or shutdown during the birth. Furthermore, the majority of autistic participants felt that their meltdown or shutdown was not handled optimally by professionals. This highlights the need for professionals to understand how to identify meltdowns and shutdowns among autistic patients and how to respond appropriately. While 17% of the non-autistic group reported a meltdown, only 8% reported shutting down during birth, perhaps indicating that non-autistic people are more likely to externalise than internalise their distress during childbirth. The opposite may be the case for autistic people (38% of whom reported shutting down vs. 29% who reported a meltdown). If this is the case, it would be important for professionals to be aware that distress during childbirth may be expressed differently by autistic and non-autistic patients. Having an advocate present during childbirth may be particularly important for autistic people given issues of communication (Donovan, 2020) and the possibility that shutting down during childbirth may make communication additionally challenging (as alluded to in the autistic participants’ qualitative responses). Indeed, the majority of autistic participants felt favourably about having an advocate present during the birth (as well as during postnatal appointments). For those who did not have an advocate, the autistic group were more likely than the non-autistic group to feel that an advocate would have been beneficial.

Autistic participants had less favourable perceptions of postnatal healthcare than non-autistic participants. For example, while the autistic group were no less likely to attend postnatal appointments, they were more likely to find postnatal home visits stressful. This could potentially be due to worrying that their home would be judged by professionals, given that autistic people can feel judged by healthcare professionals (Pohl et al., 2020; Rogers et al., 2017). Continuity of care during postnatal appointments was more likely to be considered important by the autistic group than the non-autistic group. Ensuring continuity of care may be an important adjustment that would improve postnatal healthcare for autistic people.

Participants tended to feel that professionals did not have a good understanding of autism during postnatal appointments. These findings fit with evidence that healthcare professionals can lack knowledge about autism (Morris et al., 2019; Urbanowicz et al., 2020) and indicates the need for greater autism-related training for maternity care professionals. This lack of knowledge may be a barrier to the provision of adequate healthcare for autistic people and may have influenced the fact that participants were often not offered autism-related adjustments during postnatal appointments. However, some participants may not have received adjustments due to not having received a diagnosis of autism or not having disclosed their diagnosis. Indeed, participants tended not to disclose their autism diagnosis during postnatal appointments, echoing prior findings that autistic mothers can be reluctant to disclose their diagnosis (Gardner et al., 2016; Pohl et al., 2020). Participants may have chosen not to disclose for fear of negative attitudes from professionals. Indeed, autistic participants were more likely to feel judged by and unable to trust professionals, and less likely to feel treated with respect in postnatal appointments. Whether or not participants disclosed their diagnosis did not predict responses to the postnatal healthcare experiences questions, perhaps indicating that disclosing a diagnosis does not impact upon autistic people’s experiences of postnatal healthcare. However, it is possible that non-significant results may be due to the small sample of those who did disclose; the issue of the impact of disclosure should be explored in larger samples.

Autistic participants were less likely to have received all the information they would have liked and less likely to be satisfied with how information was presented to them. This suggests the need for communication related adjustments for autistic people, such as being given the option of a variety of information formats (such as written or video formats), given difficulties processing verbal information (Nicolaidis et al., 2015; Raymaker et al., 2017). This need to make communication adjustments for autistic patients fits with prior findings that autistic people experience communication related barriers to healthcare (Nicolaidis et al., 2015; Raymaker et al., 2017) including maternity care (Donovan, 2020; Rogers et al., 2017). It also fits with findings that those with disabilities are less likely to be spoken to by professionals in a way they can understand in postnatal appointments (Malouf et al., 2017; Redshaw et al., 2013).

Adjustments to breastfeeding support may also be beneficial for autistic people. Similar to prior findings (Pohl et al., 2020), autistic participants were no less likely to breastfeed (in fact they were slightly more likely to breastfeed than non-autistic participants), though unlike prior findings (Pohl et al., 2020) autistic and non-autistic participants were just as likely to have difficulties breastfeeding. Autistic participants were, however, more likely to have difficulties breastfeeding due to sensory issues, as well as being less likely to find it easy to access breastfeeding support and less likely to feel satisfied with support they received. This fits with prior findings that people with disabilities are less likely to receive feeding support (Malouf et al., 2017).

Consistent with prior findings of increased risk of postnatal depression among autistic mothers (Pohl et al., 2020), the autistic group were more likely to experience postnatal depression and anxiety. It is worth noting that it is not clear how the levels of postnatal depression and anxiety reported relate to participants’ levels of depression and anxiety outside of the perinatal period; future longitudinal studies could tease apart these issues. Greater risk of postnatal depression and anxiety may in part be due to an increased prevalence of mental health difficulties among autistic people compared with the general population (Lai et al., 2019) and may also be influenced by additional stressors such as lower satisfaction with maternity care and sensory stressors during childbirth. These findings indicate the need for greater monitoring of, and support for, postnatal mental health among autistic people.

Autistic people may also receive less support from informal sources such as partners, friends and family. Peer support from other autistic parents may be beneficial for autistic people, with peer support being desired by the majority of participants though only available for a minority.

## Limitations

Sampling bias may have affected results. The study was only accessible to those able to complete an online survey and as such the perspectives of some autistic parents, including those with lower verbal ability, may not have been captured. The sample may also be unrepresentative due to the predominantly white, western backgrounds of the participants. Furthermore, many of the autistic group did not have a diagnosis of autism. These parents may differ in their experiences from those with a diagnosis, such as being treated differently by professionals and receiving fewer autism-related adjustments.

It is also not possible to determine whether the experiences represented are unique to autistic parents or common to parents with disabilities more broadly, given that a comparison group of parents with other disabilities was not included. It is possible that some group differences may be attributable to other factors such as the presence of other disabilities or gender identity, as these variables were not controlled for.

The survey relies on retrospective self-report. Participants reported on experiences that often occurred several years ago and this may affect the reliability of their reporting. There is some evidence that autobiographical memory may operate differently for autistic and non-autistic people, with autistic people sometimes recalling fewer memories than non-autistic people during autobiographical memory tasks (Crane & Goddard, 2008; Lind, 2010), and this may have affected the way that autistic and non-autistic participants responded to the survey. Retrospective reports may also be less relevant to current healthcare. Furthermore, participants reported on healthcare systems from a range of different countries, limiting the ability to draw conclusions specific to any particular system.

## Conclusions

This study identifies gaps in childbirth and postnatal healthcare for autistic people. During childbirth, there is a need for awareness among professionals about how to identify and respond to meltdowns and shutdowns, in addition to awareness of the non-normative ways that autistic people may experience and express physical sensations. Adjustments to the sensory environment should be made for autistic people during childbirth and where possible autistic people should be provided their own room on the postnatal ward due to sensory challenges.

The findings also highlight the need for adjustments to postnatal appointments. These include continuity of care, the provision of information in a variety of formats and the option of an advocate. Due to potential difficulties accessing group-based support (such as breastfeeding support, drop-in clinics and parent and baby groups), the availability of one-to-one support, smaller classes or online classes may be beneficial. Autistic people may also benefit from support surrounding the sensory challenges of breastfeeding. The findings also reveal a need for greater understanding of autism among birth-related and postnatal healthcare professionals. A lack of autism understanding among professionals may discourage autistic people from disclosing their diagnosis and may be a barrier to the implementation of autism-related adjustments. In addition, autistic people may have an increased risk of postnatal depression and anxiety, highlighting the need for effective mental health screening and support for autistic people during the postnatal period.

## Directions for Further Research

The findings of this self-report study should be built upon with research exploring healthcare professionals’ perspectives. This would help to establish the level of autism-related knowledge maternity professionals possess and the attitudes they hold towards autistic parents. Whether or not an autistic person chooses to disclose their autism diagnosis may affect the healthcare they receive. Disclosure may lead to adjustments being made and greater understanding from professionals, however it may conversely lead to negative attitudes from professionals. Explorations of maternity professionals’ perspectives may help to elucidate how professionals respond to a disclosure of an autism diagnosis. Qualitative work could explore experiences of disclosure from the perspective of autistic patients, including barriers to disclosing, and survey methods with larger samples could focus on differences in healthcare experiences between those who have and have not disclosed. The causal mechanisms underlying increased risk of postnatal depression and anxiety should also be addressed, with both qualitative and quantitative studies assessing the role of potential predictors such as healthcare-related stressors, socio-economic factors, level of social support, hormonal factors and prior mental health history. Further research should also explore the perinatal experiences of those aspects of the autistic community commonly neglected in research by including samples with greater representation of different ethnicities, as well as non-speaking autistic people and those with intellectual disability.

## Supplementary Information

Below is the link to the electronic supplementary material.

Supplementary file1 (DOCX 72 kb) ESM_1: Tables showing descriptive statistics for the diagnosed and self-identifying autistic groups

Supplementary file2 (DOCX 49 kb) ESM_2: Tables showing correlations between items on the survey

Supplementary file3 (DOCX 75 kb) ESM_3: Tables showing results of statistical analyses for selected findings

Supplementary file4 (DOCX 46 kb) ESM_4: Results of analyses exploring the impact of disclosure on postnatal healthcare

## Data Availability

The anonymised dataset is available on reasonable request from the corresponding author.
