# Supplementary material for: Autistic People’s Perinatal Experiences II: A Survey of Childbirth and Postnatal Experiences
Source: J Autism Dev Disord. 2022 Apr 20;53(7):2749–63. doi: 10.1007/s10803-022-05484-4 (PMC10290578; doi:10.1007/s10803-022-05484-4)
Supplement: Supplementary file 1 — Supplementary file1 (DOCX 72 kb) ESM_1: Tables showing descriptive statistics for the diagnosed and self-identifying autistic groups [file 10803_2022_5484_MOESM1_ESM.docx]

# Descriptive statistics for the diagnosed and self-identifying autistic groups

# Article title: Autistic people’s perinatal experiences II: a survey of childbirth and postnatal experiences

Journal: Journal of Autism and Developmental Disorders

Authors: Hampton, S., Allison, C., Baron-Cohen, S. & Holt, R.

Corresponding author: Sarah Hampton

Supplementary Table 1 Delivery type and gestational age

|  | Diagnosed autistic (n=224) | Self-identify as autistic (n=149) |
| --- | --- | --- |
| Delivery type: |  |  |
| Vaginal | 137 (61%) | 105 (70%) |
| Assisted vaginal | 22 (10%) | 12 (8%) |
| Elective caesarean | 23 (10%) | 15 (10%) |
| Emergency caesarean | 41 (18%) | 16 (15%) |
| Induced | 51 (23%) | 41 (28%) |
|  | Diagnosed autistic (n=224) | Self-identify as autistic (n=149) |
| Mean gestational age at birth (days)(SD) | 274 (16.00) | 277 (13.80) |

Supplementary Table 2 Childbirth experiences

|  | Diagnosed autistic | Self-identify as autistic |
| --- | --- | --- |
| I was overwhelmed by sensory input |  |  |
| N | 223 | 149 |
| Agree | 156 (70%) | 87 (58%) |
| Disagree | 58 (26%) | 45 (30%) |
| Don’t know | 3 (1%) | 5 (3%) |
| Not applicable | 6 (3%) | 12 (8%) |
| I had access to sensory items |  |  |
| N | 223 | 147 |
| Yes | 25 (11%) | 23 (16%) |
| No | 198 (89%) | 124 (84%) |
| I found it helpful to have access to sensory items |  |  |
| N | 24 | 23 |
| Agree | 22 (92%) | 21 (91%) |
| Disagree | 2 (8%) | 1 (4%) |
| Don’t know | 0 (0%) | 0 (0%) |
| Not applicable | 0 (0%) | 1 (4%) |
| I would have found it helpful to have access to sensory items |  |  |
| N | 197 | 124 |
| Agree | 102 (52%) | 58 (47%) |
| Disagree | 33 (17%) | 20 (16%) |
| Don’t know | 51 (26%) | 32 (26%) |
| Not applicable | 11 (6%) | 14 (11%) |
| I felt very aware of my body’s signals and how to correctly interpret them |  |  |
| N | 219 | 146 |
| Agree | 94 (43%) | 95 (65%) |
| Disagree | 95 (43%) | 30 (21%) |
| Don’t know | 7 (3%) | 8 (5%) |
| Not applicable | 23 (11%) | 13 (9%) |
| I experienced a meltdown during the birth |  |  |
| N | 219 | 143 |
| Yes | 68 (31%) | 37 (26%) |
| No | 151 (69%) | 106 (74%) |
| I experienced a shutdown during the birth |  |  |
| N | 219 | 145 |
| Yes | 92 (42%) | 47 (32%) |
| No | 127 (58%) | 98 (68% |
| Professionals responded to the meltdown in the way I would have liked them to |  |  |
| N | 67 | 37 |
| Agree | 20 (30%) | 14 (38%) |
| Disagree | 37 (55%) | 16 (43%) |
| Don’t know | 4 (6%) | 4 (11%) |
| Not applicable | 6 (9%) | 3 (8%) |
| Professionals responded to the shutdown in the way I would have liked them to |  |  |
| N | 91 | 47 |
| Agree | 34 (37%) | 14 (30%) |
| Disagree | 40 (44%) | 23 (49%) |
| Don’t know | 9 (10%) | 6 (13%) |
| Not applicable | 8 (9%) | 4 (9%) |
| I was kept adequately informed by health professionals of what was happening |  |  |
| N | 221 | 146 |
| Agree | 115 (52%) | 86 (59%) |
| Disagree | 97 (44%) | 53 (36%) |
| Don’t know | 4 (2%) | 2 (1%) |
| Not applicable | 5 (2%) | 5 (3%) |
| Professionals listened to my requests |  |  |
| N | 221 | 145 |
| Agree | 125 (57%) | 85 (59%) |
| Disagree | 84 (38%) | 50 (35%) |
| Don’t know | 3 (1%) | 4 (3%) |
| Not applicable | 9 (4%) | 6 (4%) |
| Professionals had an accurate understanding of what I was perceiving physically |  |  |
| N | 221 | 146 |
| Agree | 82 (37%) | 64 (44%) |
| Disagree | 115 (52%) | 64 (44%) |
| Don’t know | 11 (5%) | 5 (3%) |
| Not applicable | 13 (6%) | 13 (9%) |
| I felt pressure to behave in a socially normative way |  |  |
| N | 220 | 145 |
| Agree | 144 (65%) | 89 (61%) |
| Disagree | 53 (24%) | 44 (30%) |
| Don’t know | 14 (6%) | 5 (3%) |
| Not applicable | 9 (4%) | 7 (5%) |
| I made a birth plan |  |  |
| N | 220 | 146 |
| Yes | 136 (62%) | 98 (67%) |
| No | 84 (38%) | 48 (33%) |
| Professionals took my birth plan into account |  |  |
| N | 136 | 97 |
| Agree | 66 (49%) | 55 (57%) |
| Disagree | 56 (41%) | 33 (34%) |
| Don’t know | 5 (4%) | 3 (3%) |
| Not applicable | 9 (7%) | 6 (6%) |
| I had someone to advocate for me |  |  |
| N | 219 | 145 |
| Yes | 159 (73%) | 99 (68%) |
| No | 60 (27%) | 46 (32%) |
| I found it helpful to have someone to advocate for me |  |  |
| N | 159 | 99 |
| Agree | 128 (81%) | 84 (85%) |
| Disagree | 26 (16%) | 10 (10%) |
| Don’t know | 4 (3%) | 3 (3%) |
| Not applicable | 1 (1%) | 2 (2%) |
| I would have found it helpful to have someone to advocate for me |  |  |
| N | 59 | 46 |
| Agree | 41 (70%) | 26 (57%) |
| Disagree | 7 (12%) | 6 (13%) |
| Don’t know | 6 (10%) | 6 (13%) |
| Not applicable | 5 (9%) | 8 (17%) |
| Overall, how satisfied were you with the medical care you received? |  |  |
| N | 216 | 144 |
| Satisfied | 146 (68%) | 111 (77%) |
| Dissatisfied | 69 (32%) | 30 (21%) |
| Don’t know | 1 (1%) | 3 (2%) |
| Not applicable | 0 (0%) | 0 (0%) |
| Professionals had a good understanding of how autism affected me during the birth |  |  |
| N | 221 | 146 |
| Agree | 7 (3%) | 2 (1%) |
| Disagree | 50 (23%) | 25 (17%) |
| Don’t know | 24 (11%) | 22 (15%) |
| Not applicable | 140 (63%) | 97 (66%) |

**Supplementary Table 3** Postnatal hospital stay

|  | Diagnosed autistic | Self-identify as autistic |
| --- | --- | --- |
| I found being on a shared postnatal ward overwhelming in terms of sensory input^a^ |  |  |
| N | 119 | 67 |
| Agree | 100 (84%) | 63 (94%) |
| Disagree | 17 (14%) | 4 (6%) |
| Don’t know | 0 (0%) | 0 (0%) |
| Not applicable | 2 (2%) | 0 (0%) |
| Overall, how satisfied were you with the services you received during your postnatal stay? |  |  |
| N | 218 | 140 |
| Satisfied | 125 (57%) | 65 (46%) |
| Dissatisfied | 76 (35%) | 54 (39%) |
| Don’t know | 3 (1%) | 1 (1%) |
| Not applicable | 14 (6%) | 20 (14%) |

Supplementary Table 4 Postnatal physical and mental health

|  | Diagnosed autistic | Self-identify as autistic |
| --- | --- | --- |
| I felt prepared to cope with physical postnatal symptoms after giving birth |  |  |
| N | 218 | 139 |
| Agree | 116 (53%) | 85 (61%) |
| Disagree | 101 (46%) | 51 (37%) |
| Don’t know | 0 (0%) | 2 (1%) |
| Not applicable | 1 (1%) | 1 (1%) |
| I have known when to seek help with physical postnatal symptoms |  |  |
| N | 218 | 139 |
| Agree | 132 (61%) | 83 (60%) |
| Disagree | 79 (36%) | 53 (38%) |
| Don’t know | 5 (2%) | 2 (1%) |
| Not applicable | 2 (1%) | 1 (1%) |
| Were you told by a medical/health professional that you had postnatal depression? |  |  |
| N | 217 | 138 |
| Yes | 60 (28%) | 48 (35%) |
| No | 157 (72%) | 90 (65%) |
| Were you told by a medical/health professional that you had postnatal anxiety? |  |  |
| N | 217 | 139 |
| Yes | 34 (16%) | 35 (25%) |
| No | 183 (84%) | 104 (75%) |

Supplementary Table 5 Breastfeeding experiences

|  | Diagnosed autistic | Self-identify as autistic |
| --- | --- | --- |
| Did you breastfeed or attempt to breastfeed? |  |  |
| N | 218 | 139 |
| Yes | 207 (95%) | 130 (94%) |
| No | 11 (5%) | 9 (6%) |
| I had difficulties breastfeeding |  |  |
| N | 207 | 130 |
| Agree | 117 (57%) | 85 (65%) |
| Disagree | 89 (43%) | 45 (35%) |
| Don’t know | 0 (0%) | 0 (0%) |
| Not applicable | 1 (1%) | 0 (0%) |
| I had difficulties breastfeeding due to sensory issues |  |  |
| N | 117 | 85 |
| Agree | 60 (51%) | 34 (40%) |
| Disagree | 51 (44%) | 43 (51%) |
| Don’t know | 2 (2%) | 4 (5%) |
| Not applicable | 4 (3%) | 4 (5%) |
| I found it easy to access breastfeeding support |  |  |
| N | 206 | 130 |
| Agree | 99 (48%) | 61 (47%) |
| Disagree | 82 (40%) | 54 (42%) |
| Don’t know | 3 (2%) | 6 (5%) |
| Not applicable | 22 (11%) | 9 (7%) |
| Overall, how satisfied are you with the breastfeeding support you have received? |  |  |
| N | 207 | 130 |
| Satisfied | 94 (45%) | 67 (52%) |
| Dissatisfied | 83 (40%) | 45 (35%) |
| Don’t know | 4 (2%) | 5 (4%) |
| Not applicable | 26 (13%) | 13 (10%) |

Supplementary Table 6 Autism disclosure, adjustments and autism understanding at postnatal appointments

|  | Diagnosed autistic | Self-identify as autistic |
| --- | --- | --- |
| Disclosed autism to: |  |  |
| Midwife |  |  |
| N | 213 | 137 |
| Yes | 22 (10%) | 3 (2%) |
| No | 42 (20%) | 48 (35%) |
| Not applicable | 149 (70%) | 86 (63%) |
| Health visitor |  |  |
| N | 213 | 137 |
| Yes | 27 (13%) | 2 (2%) |
| No | 45 (21%) | 46 (34%) |
| Not applicable | 141 (66%) | 89 (65%) |
| Doctor/GP |  |  |
| N | 215 | 137 |
| Yes | 38 (18%) | 2 (2%) |
| No | 45 (21%) | 49 (36%) |
| Not applicable | 132 (61%) | 86 (63%) |
| Adjustments offered |  |  |
| N | 44 | 6 |
| Yes | 15 (34%) | 2 (33%) |
| No | 29 (66%) | 4 (67%) |
| Not applicable | 0 (0%) | 0 (0%) |
| Adjustments desired that were not offered |  |  |
| N | 44 | 6 |
| Yes | 20 (45%) | 3 (50%) |
| No | 24 (55%) | 3 (50%) |
| Not applicable | 0 (0%) | 0 (0%) |
| Health professionals have had a good understanding of how being autistic affects me: |  |  |
| Midwife |  |  |
| N | 213 | 135 |
| Agree | 13 (6%) | 12 (8%) |
| Disagree | 21 (10%) | 11 (8%) |
| Don’t know | 24 (11%) | 14 (10%) |
| Not applicable | 155 (73%) | 98 (73%) |
| Health visitor |  |  |
| N | 211 | 135 |
| Agree | 16 (8%) | 6 (4%) |
| Disagree | 24 (11%) | 14 (10%) |
| Don’t know | 23 (11%) | 18 (13%) |
| Not applicable | 148 (70%) | 97 (72%) |
| Doctor/GP |  |  |
| N | 214 | 135 |
| Agree | 21 (10%) | 10 (7%) |
| Disagree | 36 (17%) | 13 (10%) |
| Don’t know | 30 (14%) | 16 (12%) |
| Not applicable | 127 (59%) | 96 (71%) |

Supplementary Table 7 Attending postnatal appointments

|  | Diagnosed autistic | Self-identify as autistic |
| --- | --- | --- |
| Attended all |  |  |
| midwife appointments |  |  |
| N | 214 | 138 |
| Yes | 170 (79%) | 104 (75%) |
| No | 3 (1%) | 6 (4%) |
| Not applicable | 41 (19%) | 28 (20%) |
| Attended all health visitor appointments |  |  |
| N | 214 | 136 |
| Yes | 174 (81%) | 98 (72%) |
| No | 10 (5%) | 10 (7%) |
| Not applicable | 30 (14%) | 28 (21%) |
| Attended mother’s 6 week check |  |  |
| N | 215 | 137 |
| Yes | 203 (94%) | 126 (92%) |
| No | 2 (1%) | 6 (4%) |
| Not applicable | 10 (5%) | 5 (4%) |
| Attended baby’s 6-8 week check |  |  |
| N | 215 | 138 |
| Yes | 209 (97%) | 132 (96%) |
| No | 1 (1%) | 4 (3%) |
| Not applicable | 5 (2%) | 2 (1%) |

Supplementary Table 8 Postnatal appointments

|  | Diagnosed autistic | Self-identify as autistic |
| --- | --- | --- |
| I have found it stressful to have health professionals visit my home |  |  |
| N | 121 | 83 |
| Agree | 79 (65%) | 49 (59%) |
| Disagree | 40 (33%) | 34 (41%) |
| Don’t know | 2 (2%) | 0 (0%) |
| Not applicable | 0 (0%) | 0 (0%) |
| I have seen the same professional at each postnatal appointment |  |  |
| N | 212 | 136 |
| Yes | 85 (40%) | 49 (36%) |
| No | 127 (60%) | 87 (64%) |
| It is very important to me to see the same health professional at each postnatal appointment |  |  |
| N | 214 | 136 |
| Agree | 191 (89%) | 121 (89%) |
| Disagree | 17 (8%) | 8 (6%) |
| Don’t know | 6 (3%) | 4 (3%) |
| Not applicable | 0 (0%) | 3 (2%) |
| I found it stressful when the health professional I saw was not the person I was expecting to see |  |  |
| N | 212 | 136 |
| Agree | 122 (58%) | 84 (62%) |
| Disagree | 21 (10%) | 12 (9%) |
| Don’t know | 7 (3%) | 4 (3%) |
| Not applicable | 62 (29%) | 36 (26%) |
| I feel that professionals have taken seriously any questions or concerns I have had |  |  |
| N | 208 | 136 |
| Agree | 126 (61%) | 76 (56%) |
| Disagree | 72 (35%) | 51 (38%) |
| Don’t know | 4 (2%) | 3 (2%) |
| Not applicable | 6 (3%) | 6 (4%) |
| I felt comfortable asking questions to professionals |  |  |
| N | 207 | 134 |
| Agree | 127 (61%) | 71 (53%) |
| Disagree | 77 (37%) | 57 (43%) |
| Don’t know | 2 (1%) | 3 (2%) |
| Not applicable | 1 (1%) | 3 (2%) |
| Professionals have treated me respectfully |  |  |
| N | 208 | 135 |
| Agree | 149 (72%) | 94 (70%) |
| Disagree | 56 (27%) | 35 (26%) |
| Don’t know | 2 (1%) | 4 (3%) |
| Not applicable | 1 (1%) | 2 (1%) |
| I have felt negatively judged by professionals |  |  |
| N | 207 | 135 |
| Agree | 96 (46%) | 71 (53%) |
| Disagree | 96 (46%) | 56 (42%) |
| Don’t know | 6 (3%) | 5 (4%) |
| Not applicable | 9 (4%) | 3 (2%) |
| I have felt able to trust professionals |  |  |
| N | 207 | 135 |
| Agree | 121 (58%) | 72 (53%) |
| Disagree | 81 (39%) | 60 (44%) |
| Don’t know | 3 (2%) | 1 (1%) |
| Not applicable | 2 (1%) | 2 (2%) |
| I have received enough information about my mental health |  |  |
| N | 213 | 136 |
| Agree | 82 (39%) | 43 (32%) |
| Disagree | 111 (52%) | 75 (55%) |
| Don’t know | 7 (3%) | 6 (4%) |
| Not applicable | 13 (6%) | 12 (9%) |
| I have received enough information about looking after my baby |  |  |
| N | 212 | 136 |
| Agree | 69 (33%) | 35 (26%) |
| Disagree | 116 (55%) | 79 (58%) |
| Don’t know | 12 (6%) | 3 (2%) |
| Not applicable | 15 (7%) | 19 (14%) |
| I have received enough information about interpreting my baby’s cries |  |  |
| N | 211 | 136 |
| Agree | 131 (62%) | 72 (53%) |
| Disagree | 60 (28%) | 49 (36%) |
| Don’t know | 5 (2%) | 1 (1%) |
| Not applicable | 15 (7%) | 14 (10%) |
| I have received enough information about how to play with my baby |  |  |
| N | 213 | 136 |
| Agree | 75 (35%) | 44 (32%) |
| Disagree | 111 (52%) | 70 (52%) |
| Don’t know | 6 (3%) | 1 (1%) |
| Not applicable | 21 (10%) | 21 (15%) |
| I am satisfied with the way in which information was presented to me |  |  |
| N | 214 | 136 |
| Agree | 129 (60%) | 74 (54%) |
| Disagree | 71 (33%) | 51 (38%) |
| Don’t know | 8 (4%) | 4 (3%) |
| Not applicable | 6 (3%) | 7 (5%) |
| I have had someone to advocate for me during postnatal appointments |  |  |
| N | 212 | 136 |
| Yes | 101 (48%) | 56 (41%) |
| No | 111 (52%) | 80 (59%) |
| I have found it helpful to have someone to advocate for me during postnatal appointments |  |  |
| N | 101 | 55 |
| Agree | 86 (85%) | 47 (86%) |
| Disagree | 7 (7%) | 4 (7%) |
| Don’t know | 3 (3%) | 4 (7%) |
| Not applicable | 5 (5%) | 0 (0%) |
| I would have found it helpful to have someone to advocate for me during postnatal appointments |  |  |
| N | 109 | 80 |
| Agree | 65 (60%) | 43 (54%) |
| Disagree | 16 (15%) | 11 (14%) |
| Don’t know | 13 (12%) | 16 (20%) |
| Not applicable | 15 (14%) | 10 (13%) |
| Satisfaction with midwife appointments |  |  |
| N | 207 | 135 |
| Satisfied | 116 (56%) | 88 (65%) |
| Dissatisfied | 39 (19%) | 16 (12%) |
| Don’t know | 4 (2%) | 3 (2%) |
| Not applicable | 48 (23%) | 28 (21%) |
| Satisfaction with health visitor appointments |  |  |
| N | 208 | 134 |
| Satisfied | 114 (55%) | 59 (44%) |
| Dissatisfied | 50 (24%) | 38 (28%) |
| Don’t know | 5 (2%) | 5 (4%) |
| Not applicable | 39 (19%) | 32 (24%) |
| Satisfaction with doctor/GP appointments |  |  |
| N | 209 | 135 |
| Satisfied | 136 (65%) | 76 (56%) |
| Dissatisfied | 58 (28%) | 43 (32%) |
| Don’t know | 7 (3%) | 3 (2%) |
| Not applicable | 8 (4%) | 13 (10%) |
| I have found it difficult to attend drop-in clinics to get my baby weighed |  |  |
| N | 210 | 135 |
| Agree | 106 (50%) | 62 (46%) |
| Disagree | 57 (27%) | 42 (31%) |
| Don’t know | 4 (2%) | 1 (1%) |
| Not applicable | 43 (21%) | 30 (22%) |
| I have found it difficult to attend parent and baby groups |  |  |
| N | 211 | 135 |
| Agree | 175 (83%) | 102 (76%) |
| Disagree | 26 (12%) | 18 (13%) |
| Don’t know | 3 (1%) | 0 (0%) |
| Not applicable | 7 (3%) | 15 (11%) |

Supplementary Table 9 Postnatal support

|  | Diagnosed autistic | Self-identify as autistic |
| --- | --- | --- |
| I have received all the support with being a parent to my baby that I have needed from: |  |  |
| Partner/spouse |  |  |
| N | 206 | 135 |
| Agree | 108 (52%) | 70 (52%) |
| Disagree | 86 (42%) | 61 (45%) |
| Don’t know | 2 (1%) | 0 (0%) |
| Not applicable | 10 (5%) | 4 (3%) |
| Family |  |  |
| N | 205 | 135 |
| Agree | 94 (46%) | 55 (41%) |
| Disagree | 101 (49%) | 74 (55%) |
| Don’t know | 1 (1%) | 2 (2%) |
| Not applicable | 9 (4%) | 4 (3%) |
| Friends |  |  |
| N | 206 | 135 |
| Agree | 88 (43%) | 54 (40%) |
| Disagree | 89 (43%) | 61 (45%) |
| Don’t know | 2 (1%) | 4 (3%) |
| Not applicable | 27 (13%) | 16 (12%) |
| I had peer support from other autistic parents |  |  |
| N | 205 | 127 |
| Agree | 172 (84%) | 104 (82%) |
| Disagree | 33 (16%) | 23 (18%) |
| Don’t know | - | - |
| Not applicable | - | - |
| I have found it helpful to have peer support from other autistic parents |  |  |
| N | 32 | 23 |
| Agree | 31 (97%) | 23 (100%) |
| Disagree | 0 (0%) | 0 (0%) |
| Don’t know | 1 (3%) | 0 (0%) |
| Not applicable | 0 (0%) | 0 (0%) |
| I would have found it helpful to have peer support from other autistic parents |  |  |
| N | 171 | 104 |
| Agree | 114 (67%) | 51 (49%) |
| Disagree | 7 (4%) | 9 (9%) |
| Don’t know | 23 (13%) | 22 (21%) |
| Not applicable | 27 (16%) | 22 (21%) |
