# Supplementary material for: Autistic People’s Perinatal Experiences II: A Survey of Childbirth and Postnatal Experiences
Source: J Autism Dev Disord. 2022 Apr 20;53(7):2749–63. doi: 10.1007/s10803-022-05484-4 (PMC10290578; doi:10.1007/s10803-022-05484-4)
Supplement: Supplementary file 2 — Supplementary file2 (DOCX 49 kb) ESM_2: Tables showing correlations between items on the survey [file 10803_2022_5484_MOESM2_ESM.docx]

**Correlations between survey questions**

Article title: Autistic people’s perinatal experiences II: a survey of childbirth and postnatal experiences

Journal: Journal of Autism and Developmental Disorders

Authors: Hampton, S., Allison, C., Baron-Cohen, S. & Holt, R.

Corresponding author: Sarah Hampton

**Supplementary Table 1** Polychoric correlations between birth questions

|  | 1 | 2 | 3 | 4 | 5 | 6 | 7 | 8 | 9 | 10 | 11 |
| --- | --- | --- | --- | --- | --- | --- | --- | --- | --- | --- | --- |
| 1. Overwhelmed by sensory input | 1.00 |  |  |  |  |  |  |  |  |  |  |
| 2. Aware of body’s signals during birth | -.28^***^ | 1.00 |  |  |  |  |  |  |  |  |  |
| 3. Had meltdown during birth | **.44**^***^ | -.21^**^ | 1.00 |  |  |  |  |  |  |  |  |
| 4. Had shutdown during birth | **.50**^***^ | -.16^*^ | .27^***^ | 1.00 |  |  |  |  |  |  |  |
| 5. Kept informed professionals of what was happening | **-.53**^***^ | .22^***^ | **-.35**^***^ | **-.42**^***^ | 1.00 |  |  |  |  |  |  |
| 6. Professionals listened to my requests | **-.55**^***^ | **.30**^***^ | **-.36**^***^ | **-.42**^***^ | **.78**^***^ | 1.00 |  |  |  |  |  |
| 7. Professionals had accurate understanding of what perceiving physically | **-.58**^***^ | **.31**^***^ | **-.38**^***^ | **-.41**^***^ | **.75**^***^ | **.78**^***^ | 1.00 |  |  |  |  |
| 8. Felt pressure to behave in a socially normative way | **.56**^***^ | **-.32**^***^ | .16^**^ | **.36**^***^ | **-.44**^***^ | **-.47**^***^ | **-.49**^***^ | 1.00 |  |  |  |
| 9. Satisfaction with medical care received | **-.51**^***^ | .26^***^ | **-.35**^***^ | **-.45**^***^ | **.78**^***^ | **.85**^***^ | **.68**^***^ | **-.39**^***^ | 1.00 |  |  |
| 10. Shared postnatal ward overwhelming | **.44**^***^ | -.07 | .27^***^ | .24^**^ | -.21^**^ | -.21^**^ | **-.32**^***^ | .29^***^ | -.29^**^ | 1.00 |  |
| 11. Satisfaction with services received during postnatal stay | **-.32**^***^ | .21^***^ | -.18^**^ | -.19^**^ | .27^***^ | **.39**^***^ | **.33**^***^ | -.21^***^ | **.55**^***^ | **-.47**^***^ | 1.00 |

*Note.* Correlations ≥ .30 in bold

^*^p≤0.05

^**^p≤0.01

^***^p≤0.001

**Supplementary Table 2** Polychoric correlations between postnatal questions

|  | **1** | **2** | **3** | **4** | **5** | **6** | **7** | **8** | **9** | **10** | **11** | **12** | **13** | **14** | **15** | **16** | **17** | **18** | **19** | **20** | **21** | **22** | **23** |
| --- | --- | --- | --- | --- | --- | --- | --- | --- | --- | --- | --- | --- | --- | --- | --- | --- | --- | --- | --- | --- | --- | --- | --- |
| 1. Attended midwife appointments | 1.00 |  |  |  |  |  |  |  |  |  |  |  |  |  |  |  |  |  |  |  |  |  |  |
| 2. Attended health visitor appointments | **.56^***^** | 1.00 |  |  |  |  |  |  |  |  |  |  |  |  |  |  |  |  |  |  |  |  |  |
| 3. Attended 6 week check | **.72^***^** | **.58^***^** | 1.00 |  |  |  |  |  |  |  |  |  |  |  |  |  |  |  |  |  |  |  |  |
| 4. Attended 6-8 week check | **.42^*^** | **.61^**^** | **.58^***^** | 1.00 |  |  |  |  |  |  |  |  |  |  |  |  |  |  |  |  |  |  |  |
| 5. Seen the same professional at each appointment | -.06 | .08 | **.58** | **.33^***^** | 1.00 |  |  |  |  |  |  |  |  |  |  |  |  |  |  |  |  |  |  |
| 6. Seeing the same professional at each appointment is important | **-.27** | .01 | -.21 | -.19^*^ | .28^***^ | 1.00 |  |  |  |  |  |  |  |  |  |  |  |  |  |  |  |  |  |
| 7. Stressful when professional not who expecting | -.14 | -.06 | -.14 | -.24 | -.03 | **.79^***^** | 1.00 |  |  |  |  |  |  |  |  |  |  |  |  |  |  |  |  |
| 8. Found home visits stressful | -.16 | -.22 | -.05 | -.16 | -.12 | **.30^***^** | **.52^***^** | 1.00 |  |  |  |  |  |  |  |  |  |  |  |  |  |  |  |
| 9. Professionals took concerns seriously | .01 | .16 | **.38^***^** | **.31^*^** | **.35^***^** | -.15^*^ | **-.38^***^** | **-.39^**^** | 1.00 |  |  |  |  |  |  |  |  |  |  |  |  |  |  |
| 10. Professionals have treated me respectfully | .13 | .22^*^ | .16 | .20 | .26^***^ | -.18^*^ | **-.37^***^** | **-.44^***^** | **.78^***^** | 1.00 |  |  |  |  |  |  |  |  |  |  |  |  |  |
| 11. I have felt able to trust professionals | .02 | .29^***^ | .27^**^ | .19 | .26^***^ | -.22^***^ | **-.43^***^** | **-.46^***^** | **.76^***^** | **.82^***^** | 1.00 |  |  |  |  |  |  |  |  |  |  |  |  |
| 12. Comfortable asking questions to professionals | -.03 | .25^**^ | .22^*^ | **.30^*^** | .29^***^ | -.28^***^ | **-.52^***^** | **-.47^***^** | **.83^***^** | **.82^***^** | **.80^***^** | 1.00 |  |  |  |  |  |  |  |  |  |  |  |
| 13. I have felt negatively judged by professionals | -.24 | -.28^**^ | -.23^*^ | **-.37^*^** | -.27^***^ | .24^***^ | **.43^***^** | **.50^***^** | **-.62^***^** | **-.70^***^** | **-.68^***^** | **-.59^***^** | 1.00 |  |  |  |  |  |  |  |  |  |  |
| 14. Received information on mental health | .16 | .18 | .21 | -.04 | .13^*^ | -.19^***^ | **-.37^***^** | -.19^*^ | **.50^***^** | **.50^***^** | **.55^***^** | **.58^***^** | **-.41^***^** | 1.00 |  |  |  |  |  |  |  |  |  |
| 15. Received information on looking after baby | .13 | .29^*^ | .11 | .09 | .11^*^ | -.13 | -.25^***^ | -.13 | **.42^***^** | **.36^***^** | **.33^***^** | **.46^***^** | -.18 | **.60^***^** | 1.00 |  |  |  |  |  |  |  |  |
| 16. Received information on interpreting baby’s cries | .11 | .26^**^ | .16 | **.31^*^** | .09 | -.18^*^ | -.26^***^ | -.13 | **.43^***^** | **.41^***^** | **.43^***^** | **.53^***^** | -.28^***^ | **.61^***^** | **.79^***^** | 1.00 |  |  |  |  |  |  |  |
| 17. Received information on play with baby | .13 | .15 | -.05 | .06 | .04 | -.08 | -.19^*^ | -.14 | .28^***^ | .28^***^ | .22^***^ | **.33^***^** | -.10^***^ | **.56^***^** | **.88^***^** | **.77^***^** | 1.00 |  |  |  |  |  |  |
| 18. Satisfied how information presented | .09 | **.32^**^** | **.32^*^** | .29 | .21^***^ | -.25^***^ | **-.32^***^** | -.24^***^ | **.73^***^** | **.64^***^** | **.69^***^** | **.75^***^** | **-.46^***^** | **.69^***^** | **.67^***^** | **.70^***^** | **.59^***^** | 1.00 |  |  |  |  |  |
| 19. Satisfaction midwife appointments | .02 | .11 | .21 | .06 | **.32^***^** | -.27^***^ | **-.36^**^** | **-.44^**^** | **.67^***^** | **.66^***^** | **.76^***^** | **.69^***^** | **-.51^***^** | **.50^***^** | **.44^***^** | **.54^***^** | **.37^***^** | **.69^***^** | 1.00 |  |  |  |  |
| 20. Satisfaction health visitor appointments | .07 | **.58^***^** | .14 | **.43^**^** | .24^***^ | -.15 | -.24^**^ | **-.38^***^** | **.51^***^** | **.68^***^** | **.67^***^** | **.65^***^** | **-.59^***^** | **.33^***^** | **.33^***^** | **.41^***^** | .24^***^ | **.58^***^** | **.58^***^** | 1.00 |  |  |  |
| 21. Satisfaction doctor appointments | .06 | .17 | .26 | .23 | .26^**^ | -.20^**^ | **-.32^**^** | -.19^**^ | **.64^***^** | **.65^***^** | **.63^***^** | **.56^***^** | **-.43^***^** | **.42^***^** | **.32^***^** | **.37^***^** | .25^**^ | **.60^***^** | **.59^***^** | **.58^***^** | 1.00 |  |  |
| 22. Difficult to attend drop-in clinics | -.04 | -.24^*^ | -.19 | **-.50^***^** | -.08 | **.31^***^** | **.45^**^** | **.30^***^** | **-.36^***^** | **-.38^***^** | **-.31^***^** | **-.47^***^** | .29^***^ | -.19^***^ | -.20^***^ | -.24^***^ | -.15^**^ | **-.34^***^** | -.13^*^ | -.24^***^ | **-.35^***^** | 1.00 |  |
| 23. Difficult to attend parent and baby groups | -.18 | -.13 | -.24 | -.16 | .10 | **.34^***^** | **.52^***^** | **.54^***^** | **-.36^***^** | **-.32^***^** | **-.38^***^** | **-.45^***^** | **.38^***^** | **-.38^***^** | **-.31^**^** | -.21^**^ | -.27^***^ | -.29^***^ | -.29^***^ | -.20^**^ | -.25^***^ | **.58^***^** | 1.00 |

*Note.* Correlations ≥ .30 in bold

^*^p≤0.05

^**^p≤0.01

^***^p≤0.001

**Supplementary Table 3** Polychoric correlations between breastfeeding questions

|  | Had difficulties breastfeeding | Found it easy to access breastfeeding support | Satisfaction with breastfeeding support |
| --- | --- | --- | --- |
| Had difficulties breastfeeding | 1.00 |  |  |
| Found it easy to access breastfeeding support | **-.41**^***^ | 1.00 |  |
| Satisfaction with breastfeeding support | **-.52**^***^ | **.90**^***^ | 1.00 |

*Note.* Correlations ≥ .30 in bold

^***^p≤0.001

**Supplementary Table 4** Polychoric correlations between postnatal support questions

|  | Partner | Family | Friends |
| --- | --- | --- | --- |
| Partner | 1.00 |  |  |
| Family | **.58**^***^ | 1.00 |  |
| Friends | **.37**^***^ | **.66**^***^ | 1.00 |

*Note.* Correlations ≥ .30 in bold

^***^p≤0.001
