# Supplementary material for: Autistic People’s Perinatal Experiences II: A Survey of Childbirth and Postnatal Experiences
Source: J Autism Dev Disord. 2022 Apr 20;53(7):2749–63. doi: 10.1007/s10803-022-05484-4 (PMC10290578; doi:10.1007/s10803-022-05484-4)
Supplement: Supplementary file 3 — Supplementary file3 (DOCX 75 kb) ESM_3: Tables showing results of statistical analyses for selected findings [file 10803_2022_5484_MOESM3_ESM.docx]

# Results of statistical analyses for selected findings

# Article title: Autistic people’s perinatal experiences II: a survey of childbirth and postnatal experiences

Journal: Journal of Autism and Developmental Disorders

Authors: Hampton, S., Allison, C., Baron-Cohen, S. & Holt, R.

Corresponding author: Sarah Hampton

Supplementary Table 1 Childbirth experiences

|  | Non-autistic group | Autistic group | aOR (95% CI) | | p-value | p-value (FDR adjusted) | |  |  |
| --- | --- | --- | --- | --- | --- | --- | --- | --- | --- |
| I was overwhelmed by sensory input^a^ |  |  | 7.63 (5.08 - 11.44) | | **<0.001** | **-** | |  |  |
| N | 479 | 372 |  | |  |  | |  |  |
| Agree | 140 (29%) | 243 (65%) |  | |  |  | |  |  |
| Disagree | 310 (65%) | 103 (28%) |  | |  |  | |  |  |
| Don’t know | 4 (1%) | 8 (2%) |  | |  |  | |  |  |
| Not applicable | 25 (5%) | 18 (5%) |  | |  |  | |  |  |
| I had access to sensory items^b^ |  |  | 1.09 (0.68 - 1.74) | | 0.72 | 0.92 | |  |  |
| N | 490 | 370 |  | |  |  | |  |  |
| Yes | 68 (14%) | 48 (13%) |  | |  |  | |  |  |
| No | 422 (86%) | 322 (87%) |  | |  |  | |  |  |
| I found it helpful to have access to sensory items^b^ |  |  | 9.02 (1.24 - 139.73) | | 0.06 | 0.14 | |  |  |
| N | 66 | 47 |  | |  |  | |  |  |
| Agree | 45 (68%) | 43 (92%) |  | |  |  | |  |  |
| Disagree | 10 (15%) | 3 (6%) |  | |  |  | |  |  |
| Don’t know | 2 (3%) | 0 (0%) |  | |  |  | |  |  |
| Not applicable | 9 (14%) | 1 (2%) |  | |  |  | |  |  |
| I would have found it helpful to have access to sensory items^b^ |  |  | 7.85 (4.95 - 12.68) | | **<0.001** | **<0.001** | |  |  |
| N | 410 | 321 |  | |  |  | |  |  |
| Agree | 70 (17%) | 160 (50%) |  | |  |  | |  |  |
| Disagree | 218 (53%) | 53 (17%) |  | |  |  | |  |  |
| Don’t know | 64 (16%) | 83 (26%) |  | |  |  | |  |  |
| Not applicable | 58 (14%) | 25 (8%) |  | |  |  | |  |  |
| I felt very aware of my body’s signals and how to correctly interpret them |  |  | 0.53 (0.35 - 0.80) | | **0.002** | **-** | |  |  |
| N | 472 | 365 |  | |  |  | |  |  |
| Agree | 306 (65%) | 189 (52%) |  | |  |  | |  |  |
| Disagree | 108 (23%) | 125 (34%) |  | |  |  | |  |  |
| Don’t know | 3 (1%) | 15 (4%) |  | |  |  | |  |  |
| Not applicable | 55 (12%) | 36 (10%) |  | |  |  | |  |  |
| I experienced a meltdown during the birth^a^ |  |  | 2.10 (1.37 - 3.22) | | **0.001** | **-** | |  |  |
| N | 485 | 362 |  | |  |  | |  |  |
| Yes | 81 (17%) | 105 (29%) |  | |  |  | |  |  |
| No | 404 (83%) | 257 (71%) |  | |  |  | |  |  |
| I experienced a shutdown during the birth^a^ |  |  | 9.80 (6.06 - 15.85) | | **<0.001** | **-** | |  |  |
| N | 485 | 364 |  | |  |  | |  |  |
| Yes | 39 (8%) | 139 (38%) |  | |  |  | |  |  |
| No | 446 (92%) | 225 (62% |  | |  |  | |  |  |
| Professionals responded to the meltdown in the way I would have liked them to^b^ |  |  | 0.42 (0.19 - 0.89) | | **0.02** | 0.07 | |  |  |
| N | 80 | 104 |  | |  |  | |  |  |
| Agree | 47 (59%) | 34 (33%) |  | |  |  | |  |  |
| Disagree | 26 (33%) | 53 (51%) |  | |  |  | |  |  |
| Don’t know | 6 (8%) | 8 (8%) |  | |  |  | |  |  |
| Not applicable | 1 (1%) | 9 (9%) |  | |  |  | |  |  |
| Professionals responded to the shutdown in the way I would have liked them to^b^ |  |  | 0.57 (0.22- 1.43) | | 0.24 | 0.46 | |  |  |
| N | 38 | 138 |  | |  |  | |  |  |
| Agree | 19 (50%) | 48 (35%) |  | |  |  | |  |  |
| Disagree | 13 (34%) | 63 (46%) |  | |  |  | |  |  |
| Don’t know | 5 (13%) | 15 (11%) |  | |  |  | |  |  |
| Not applicable | 1 (3%) | 12 (9%) |  | |  |  | |  |  |
| I was kept adequately informed by health professionals of what was happening |  |  | | 0.37 (0.25 - 0.56) | **<0.001** | | **-** | |  |
| N | 476 | 367 | |  |  | |  | |  |
| Agree | 348 (73%) | 201 (55%) | |  |  | |  | |  |
| Disagree | 108 (23%) | 150 (41%) | |  |  | |  | |  |
| Don’t know | 3 (1%) | 6 (2%) | |  |  | |  | |  |
| Not applicable | 17 (4%) | 10 (3%) | |  |  | |  | |  |
| Professionals listened to my requests |  |  | | 0.32 (0.21 -0.49) | **<0.001** | | **-** | |  |
| N | 473 | 366 | |  |  | |  | |  |
| Agree | 354 (75%) | 210 (57%) | |  |  | |  | |  |
| Disagree | 82 (17%) | 134 (37%) | |  |  | |  | |  |
| Don’t know | 8 (2%) | 7 (2%) | |  |  | |  | |  |
| Not applicable | 29 (6%) | 15 (4%) | |  |  | |  | |  |
| Professionals had an accurate understanding of what I was perceiving physically |  |  | | 0.17 (0.11 - 0.25) | **<0.001** | | **-** | |  |
| N | 475 | 367 | |  |  | |  | |  |
| Agree | 344 (72%) | 146 (40%) | |  |  | |  | |  |
| Disagree | 96 (20%) | 179 (49%) | |  |  | |  | |  |
| Don’t know | 7 (1%) | 16 (4%) | |  |  | |  | |  |
| Not applicable | 28 (6%) | 26 (7%) | |  |  | |  | |  |
| I felt pressure to behave in a socially normative way^a^ |  |  | | 5.15 (3.43 -7.81) | **<0.001** | | - | |  |
| N | 472 | 365 | |  |  | |  | |  |
| Agree | 161 (34%) | 233 (64%) | |  |  | |  | |  |
| Disagree | 261 (55%) | 97 (27%) | |  |  | |  | |  |
| Don’t know | 14 (3%) | 19 (5%) | |  |  | |  | |  |
| Not applicable | 36 (8%) | 16 (4%) | |  |  | |  | |  |
| I made a birth plan^b^ |  |  | | 1.21 (0.87 - 1.69) | 0.26 | | 0.46 | | |
| N | 484 | 366 | |  |  | |  | | |
| Yes | 293 (61%) | 234 (64%) | |  |  | |  | | |
| No | 191 (39%) | 132 (36%) | |  |  | |  | | |
| Professionals took my birth plan into account^b^ |  |  | | 0.51 (0.33 -0.79) | **0.003** | | **0.01** | | |
| N | 289 | 233 | |  |  | |  | | |
| Agree | 188 (65%) | 121 (52%) | |  |  | |  | | |
| Disagree | 69 (24%) | 89 (38%) | |  |  | |  | | |
| Don’t know | 7 (2%) | 8 (3%) | |  |  | |  | | |
| Not applicable | 25 (9%) | 15 (6%) | |  |  | |  | | |
| I had someone to advocate for me^b^ |  |  | | 0.97 (0.69 -1.38) | 0.87 | | 0.92 | | |
| N | 472 | 364 | |  |  | |  | | |
| Yes | 352 (75%) | 258 (71%) | |  |  | |  | | |
| No | 120 (25%) | 106 (29%) | |  |  | |  | | |
| I found it helpful to have someone to advocate for me^b^ |  |  | | 0.64 (0.35 -1.17) | 0.15 | | 0.33 | | |
| N | 352 | 258 | |  |  | |  | | |
| Agree | 307 (87%) | 212 (82%) | |  |  | |  | | |
| Disagree | 29 (8%) | 36 (14%) | |  |  | |  | | |
| Don’t know | 5 (1%) | 7 (3%) | |  |  | |  | | |
| Not applicable | 11 (3%) | 3 (1%) | |  |  | |  | | |
| I would have found it helpful to have someone to advocate for me^b^ |  |  | | 7.55 (3.27 -18.90) | **<0.001** | | **<0.001** | | |
| N | 120 | 105 | |  |  | |  | | |
| Agree | 40 (33%) | 67 (64%) | |  |  | |  | | |
| Disagree | 57 (48%) | 13 (12%) | |  |  | |  | | |
| Don’t know | 6 (5%) | 12 (11%) | |  |  | |  | | |
| Not applicable | 17 (14%) | 13 (12%) | |  |  | |  | | |
| Overall, how satisfied were you with the medical care you received? |  |  | | 0.39 (0.25 - 0.61) | **<0.001** | | **-** | | |
| N | 480 | 360 | |  |  | |  | | |
| Satisfied | 414 (86%) | 257 (71%) | |  |  | |  | | |
| Dissatisfied | 64 (13%) | 99 (27%) | |  |  | |  | | |
| Don’t know | 2 (0.42%) | 4 (1%) | |  |  | |  | | |
| Not applicable | 0 (0%) | 0 (0%) | |  |  | |  | | |
| Professionals had a good understanding of how autism affected me during the birth^b^ |  |  | |  |  | |  | | |
| N | - | 367 | | - | - | | - | | |
| Agree | - | 9 (2%) | | - | - | | - | | |
| Disagree | - | 75 (20%) | | - | - | | - | | |
| Don’t know | - | 46 (13%) | | - | - | | - | | |
| Not applicable | - | 237 (65%) | | - | - | | - | | |

*Note.* Multivariate binary logistic regression performed

^a^Item reverse scored prior to multivariate analysis. Inverse of aOR and CIs presented

^b^Item not included within multivariate analysis due to survey logic

Supplementary Table 2 Breastfeeding experiences

|  | Non-autistic group | Autistic group | aOR (95% CI) | p-value | p-value (FDR adjusted) |
| --- | --- | --- | --- | --- | --- |
| Did you breastfeed or attempt to breastfeed?^a^ |  |  | 2.11 (1.12 -4.09) | **0.02** | **0.03** |
| N | 435 | 357 |  |  |  |
| Yes | 397 (91%) | 337 (94%) |  |  |  |
| No | 38 (9%) | 20 (6%) |  |  |  |
| I had difficulties breastfeeding^b^ |  |  | 1.38 (0.81 - 2.34) | 0.23 | - |
| N | 397 | 337 |  |  |  |
| Agree | 227 (57%) | 202 (60%) |  |  |  |
| Disagree | 167 (42%) | 134 (40%) |  |  |  |
| Don’t know | 1 (0.25%) | 0 (0%) |  |  |  |
| Not applicable | 2 (0.50%) | 1 (0.30%) |  |  |  |
| I had difficulties breastfeeding due to sensory issues^a^ |  |  | 6.88 (3.93 -12.46) | **<0.001** | **<0.001** |
| N | 227 | 202 |  |  |  |
| Agree | 22 (10%) | 94 (47%) |  |  |  |
| Disagree | 189 (83%) | 94 (47%) |  |  |  |
| Don’t know | 4 (2%) | 6 (3%) |  |  |  |
| Not applicable | 12 (5%) | 8 (4%) |  |  |  |
| I found it easy to access breastfeeding support |  |  | 0.31 (0.18 - 0.55) | **<0.001** | **-** |
| N | 397 | 336 |  |  |  |
| Agree | 238 (60%) | 160 (48%) |  |  |  |
| Disagree | 116 (29%) | 136 (40%) |  |  |  |
| Don’t know | 5 (1%) | 9 (3%) |  |  |  |
| Not applicable | 38 (10%) | 31 (9%) |  |  |  |
| Overall, how satisfied are you with the breastfeeding support you have received? |  |  | 0.38 (0.22 -0.68) | **0.001** | **-** |
| N | 396 | 337 |  |  |  |
| Satisfied | 223 (56%) | 161 (48%) |  |  |  |
| Dissatisfied | 114 (29%) | 128 (38%) |  |  |  |
| Don’t know | 8 (2%) | 9 (3%) |  |  |  |
| Not applicable | 51 (13%) | 39 (12%) |  |  |  |

*Note.* Multivariate binary logistic regression performed

^a^Item not included within multivariate analysis due to survey logic

^b^Item reverse scored prior to multivariate analysis. Inverse of aOR and CIs presented

Supplementary Table 3 Postnatal appointments

|  | Non-autistic group | Autistic group | aOR (95% CI) | p-value | p-value (FDR adjusted) | | | |
| --- | --- | --- | --- | --- | --- | --- | --- | --- |
| I have found it stressful to have health professionals visit my home^a^ |  |  | 9.43 (5.75 - 15.46) | **<0.001** | - | | | |
| N | 295 | 204 |  |  |  | | | |
| Agree | 65 (22%) | 128 (63%) |  |  |  | | | |
| Disagree | 224 (76%) | 74 (36%) |  |  |  | | | |
| Don’t know | 3 (1%) | 2 (1%) |  |  |  | | | |
| Not applicable | 3 (1%) | 0 (0%) |  |  |  | | | |
| I have seen the same professional at each postnatal appointment |  |  | 0.94 (0.64 -1.38) | 0.75 | - | | | |
| N | 424 | 348 |  |  |  | | | |
| Yes | 169 (40%) | 134 (39%) |  |  |  | | | |
| No | 255 (60%) | 214 (61%) |  |  |  | | | |
| It is very important to me to see the same health professional at each postnatal appointment^a^ |  |  | 4.76 (2.75 - 8.20) | **<0.001** | - | | | |
| N | 426 | 350 |  |  |  | | | |
| Agree | 323 (76%) | 312 (89%) |  |  |  | | | |
| Disagree | 95 (22%) | 25 (7%) |  |  |  | | | |
| Don’t know | 2 (0.47%) | 10 (3%) |  |  |  | | | |
| Not applicable | 6 (1%) | 3 (1%) |  |  |  | | | |
| I found it stressful when the health professional I saw was not the person I was expecting to see^a^ |  |  | 14.43 (8.55 - 24.33) | **<0.001** | - | | | |
| N | 426 | 348 |  |  |  | | | |
| Agree | 131 (31%) | 206 (59%) |  |  |  | | | |
| Disagree | 183 (43%) | 33 (9%) |  |  |  | | | |
| Don’t know | 6 (1%) | 11 (3%) |  |  |  | | | |
| Not applicable | 106 (25%) | 98 (28%) |  |  |  | | | |
| I feel that professionals have taken seriously any questions or concerns I have had |  |  | 0.22 (0.14 - 0.34) | **<0.001** | | - | | |
| N | 423 | 344 |  |  | |  | | |
| Agree | 346 (82%) | 202 (59%) |  |  | |  | | |
| Disagree | 64 (15%) | 123 (36%) |  |  | |  | | |
| Don’t know | 3 (1%) | 7 (2%) |  |  | |  | | |
| Not applicable | 10 (2%) | 12 (3%) |  |  | |  | | |
| I felt comfortable asking questions to professionals |  |  | 0.14 (0.09 -0.22) | **<0.001** | | - | | |
| N | 421 | 341 |  |  | |  | | |
| Agree | 346 (85%) | 198 (58%) |  |  | |  | | |
| Disagree | 51 (12%) | 134 (39%) |  |  | |  | | |
| Don’t know | 3 (1%) | 5 (2%) |  |  | |  | | |
| Not applicable | 8 (2%) | 4 (1%) |  |  | |  | | |
| Professionals have treated me respectfully |  |  | 0.17 (0.10 - 0.29) | **<0.001** | | - | | |
| N | 421 | 343 |  |  | |  | | |
| Agree | 378 (90%) | 243 (71%) |  |  | |  | | |
| Disagree | 34 (8%) | 91 (27%) |  |  | |  | | |
| Don’t know | 2 (0.48%) | 6 (2%) |  |  | |  | | |
| Not applicable | 7 (2%) | 3 (1%) |  |  | |  | | |
| I have felt negatively judged by professionals^a^ |  |  | 4.93 (3.26 - 7.46) | **<0.001** | | - | | |
| N | 423 | 342 |  |  | |  | | |
| Agree | 97 (23%) | 167 (49%) |  |  | |  | | |
| Disagree | 302 (71%) | 152 (44%) |  |  | |  | | |
| Don’t know | 1 (0.24%) | 11 (3%) |  |  | |  | | |
| Not applicable | 23 (5%) | 12 (4%) |  |  | |  | | |
| I have felt able to trust professionals |  |  | 0.18 (0.11 -0.28) | **<0.001** | | - | | |
| N | 422 | 342 |  |  | |  | | |
| Agree | 347 (82%) | 193 (56%) |  |  | |  | | |
| Disagree | 65 (15%) | 141 (41%) |  |  | |  | | |
| Don’t know | 3 (1%) | 4 (1%) |  |  | |  | | |
| Not applicable | 7 (2%) | 4 (1%) |  |  | |  | | |
| I have received enough information about my mental health |  |  | 0.24 (0.16 -0.35) | **<0.001** | - | | | |
| N | 427 | 349 |  |  |  | | | |
| Agree | 255 (60%) | 125 (36%) |  |  |  | | | |
| Disagree | 126 (30%) | 186 (53%) |  |  |  | | | |
| Don’t know | 9 (2%) | 13 (4%) |  |  |  | | | |
| Not applicable | 37 (9%) | 25 (7%) |  |  |  | | | |
| I have received enough information about looking after my baby |  |  | 0.40 (0.26 - 0.60) | **<0.001** | - | | | |
| N | 426 | 348 |  |  |  | | | |
| Agree | 184 (43%) | 104 (30%) |  |  |  | | | |
| Disagree | 169 (40%) | 195 (56%) |  |  |  | | | |
| Don’t know | 7 (2%) | 15 (4%) |  |  |  | | | |
| Not applicable | 66 (15%) | 34 (10%) |  |  |  | | | |
| I have received enough information about interpreting my baby’s cries |  |  | 0.43 (0.28 - 0.67) | **<0.001** | - | | | |
| N | 427 | 347 |  |  |  | | | |
| Agree | 297 (70%) | 203 (59%) |  |  |  | | | |
| Disagree | 78 (18%) | 109 (31%) |  |  |  | | | |
| Don’t know | 4 (1%) | 6 (2%) |  |  |  | | | |
| Not applicable | 48 (11%) | 29 (8%) |  |  |  | | | |
| I have received enough information about how to play with my baby |  |  | 0.49 (0.32 - 0.74) | **<0.001** | - | | | |
| N | 427 | 349 |  |  |  | | | |
| Agree | 187 (44%) | 119 (34%) |  |  |  | | | |
| Disagree | 165 (39%) | 181 (52%) |  |  |  | | | |
| Don’t know | 4 (1%) | 7 (2%) |  |  |  | | | |
| Not applicable | 71 (17%) | 42 (12%) |  |  |  | | | |
| I am satisfied with the way in which information was presented to me |  |  | 0.25 (0.16 -0.38) | **<0.001** | - | | | |
| N | 427 | 350 |  |  |  | | | |
| Agree | 341 (80%) | 203 (58%) |  |  |  | | | |
| Disagree | 66 (15%) | 122 (35%) |  |  |  | | | |
| Don’t know | 5 (1%) | 12 (3%) |  |  |  | | | |
| Not applicable | 15 (4%) | 13 (4%) |  |  |  | | | |
| I have had someone to advocate for me during postnatal appointments^b^ |  |  | 0.94 (0.68 - 1.30) | 0.70 | 0.70 | | | |
| N | 421 | 348 |  |  |  | | | |
| Yes | 204 (49%) | 157 (45%) |  |  |  | | | |
| No | 217 (52%) | 191 (55%) |  |  |  | | | |
| I have found it helpful to have someone to advocate for me during postnatal appointments^b^ |  |  | 2.03 (0.85 - 5.16) | 0.12 | 0.10 | | | |
| N | 203 | 156 |  |  |  | | | |
| Agree | 160 (79%) | 133 (85%) |  |  |  | | | |
| Disagree | 21 (10%) | 11 (7%) |  |  |  | | | |
| Don’t know | 8 (4%) | 7 (5%) |  |  |  | | | |
| Not applicable | 14 (7%) | 5 (3%) |  |  |  | | | |
| I would have found it helpful to have someone to advocate for me during postnatal appointments^b^ |  |  | 6.67 (3.73 - 12.27) | **<0.001** | **<0.001** | | | |
| N | 216 | 189 |  |  |  | | | |
| Agree | 50 (23%) | 108 (57%) |  |  |  | | | |
| Disagree | 108 (50%) | 27 (14%) |  |  |  | | | |
| Don’t know | 15 (7%) | 29 (15%) |  |  |  | | | |
| Not applicable | 43 (20%) | 25 (13%) |  |  |  | | | |
| Satisfaction with midwife appointments |  |  | 0.25 (0.14 - 0.44) | **<0.001** | | | - |  |
| N | 423 | 342 |  |  | | |  |  |
| Satisfied | 328 (78%) | 204 (60%) |  |  | | |  |  |
| Dissatisfied | 29 (7%) | 55 (16%) |  |  | | |  |  |
| Don’t know | 4 (1%) | 7 (2%) |  |  | | |  |  |
| Not applicable | 62 (15%) | 76 (22%) |  |  | | |  |  |
| Satisfaction with health visitor appointments |  |  | 0.36 (0.23 - 0.58) | **<0.001** | | | - |  |
| N | 422 | 342 |  |  | | |  |  |
| Satisfied | 305 (72%) | 173 (51%) |  |  | | |  |  |
| Dissatisfied | 69 (16%) | 88 (26%) |  |  | | |  |  |
| Don’t know | 1 (0.24%) | 10 (3%) |  |  | | |  |  |
| Not applicable | 47 (11%) | 71 (21%) |  |  | | |  |  |
| Satisfaction with doctor/GP appointments |  |  | 0.30 (0.19 -0.47) | **<0.001** | | | - |  |
| N | 422 | 344 |  |  | | |  |  |
| Satisfied | 341 (81%) | 212 (62%) |  |  | | |  |  |
| Dissatisfied | 59 (14%) | 101 (29%) |  |  | | |  |  |
| Don’t know | 0 (0%) | 10 (3%) |  |  | | |  |  |
| Not applicable | 22 (5%) | 21 (6%) |  |  | | |  |  |
| I have found it difficult to attend drop-in clinics to get my baby weighed^a^ |  |  | 4.35 (2.80 - 6.66) | **<0.001** | - | | | |
| N | 422 | 345 |  |  |  | | | |
| Agree | 123 (29%) | 168 (49%) |  |  |  | | | |
| Disagree | 234 (55%) | 99 (29%) |  |  |  | | | |
| Don’t know | 3 (1%) | 5 (2%) |  |  |  | | | |
| Not applicable | 62 (15%) | 73 (21%) |  |  |  | | | |
| I have found it difficult to attend parent and baby groups^a^ |  |  | 13.25 (8.33 - 21.10) | **<0.001** | - | | | |
| N | 423 | 346 |  |  |  | | | |
| Agree | 174 (41%) | 277 (80%) |  |  |  | | | |
| Disagree | 208 (49%) | 44 (13%) |  |  |  | | | |
| Don’t know | 3 (1%) | 3 (1%) |  |  |  | | | |
| Not applicable | 38 (9%) | 22 (6%) |  |  |  | | | |

*Note.* Multivariate binary logistic regression performed

^a^Item reverse scored prior to multivariate analysis. Inverse of aOR and CIs presented

^b^Item not included within multivariate analysis due to survey logic

Supplementary Table 4 Postnatal support

|  | | | Non-autistic group | | Autistic group | | aOR (95% CI) | p-value |
| --- | --- | --- | --- | --- | --- | --- | --- | --- |
| I have received all the support with being a parent to my baby that I have needed from: | | |  | |  | |  |  |
| Partner/spouse | | |  | |  | | 0.45 (0.29 - 0.70) | **<0.001** |
| N | | | 414 | | 341 | |  |  |
| Agree | | | 300 (72%) | | 178 (52%) | |  |  |
| Disagree | | | 107 (26%) | | 147 (43%) | |  |  |
| Don’t know | | | 0 (0%) | | 2 (1%) | |  |  |
| Not applicable | | | 7 (2%) | | 14 (4%) | |  |  |
| Family | | |  | |  | | 0.31 (0.20 - 0.48) | **<0.001** |
| N | | | 414 | | 340 | |  |  |
| Agree | | | 292 (71%) | | 149 (44%) | |  |  |
| Disagree | | | 114 (28%) | | 175 (51%) | |  |  |
| Don’t know | | | 1 (0.24%) | | 3 (1%) | |  |  |
| Not applicable | | | 7 (2%) | | 13 (4%) | |  |  |
| Friends | | |  | |  | | 0.34 (0.21 - 0.53) | **<0.001** |
| N | | | 414 | | 341 | |  |  |
| Agree | | | 293 (71%) | | 142 (42%) | |  |  |
| Disagree | | | 107 (26%) | | 150 (44%) | |  |  |
| Don’t know | | | 4 (1%) | | 6 (2%) | |  |  |
| Not applicable | | | 10 (2%) | | 43 (13%) | |  |  |
|  | N | Agree | | Disagree | | Don’t know | Not applicable | |
| I had peer support from other autistic parents^b^ | 332 | 56 (17%) | | 276 (83%) | | - | - | |
| I have found it helpful to have peer support from other autistic parents^b^ | 55 | 54 (98%) | | 0 (0%) | | 1 (2%) | 0 (0%) | |
| I would have found it helpful to have peer support from other autistic parents^b^ | 275 | 165 (60%) | | 16 (6%) | | 45 (16%) | 49 (18%) | |

*Note.* Multivariate binary logistic regression performed

^b^Item not included within multivariate analysis
