# Supplementary material for: Autistic People’s Perinatal Experiences II: A Survey of Childbirth and Postnatal Experiences
Source: J Autism Dev Disord. 2022 Apr 20;53(7):2749–63. doi: 10.1007/s10803-022-05484-4 (PMC10290578; doi:10.1007/s10803-022-05484-4)
Supplement: Supplementary file 4 — Supplementary file4 (DOCX 46 kb) ESM_4: Results of analyses exploring the impact of disclosure on postnatal healthcare [file 10803_2022_5484_MOESM4_ESM.docx]

# Results of analyses exploring the impact of disclosure on postnatal healthcare

# Article title: Autistic people’s perinatal experiences II: a survey of childbirth and postnatal experiences

Journal: Journal of Autism and Developmental Disorders

Authors: Hampton, S., Allison, C., Baron-Cohen, S. & Holt, R.

Corresponding author: Sarah Hampton

In order to explore the impact of disclosure of an autism diagnosis on experiences of postnatal healthcare, a multivariate binary logistic regression was performed for questions concerning postnatal appointments, with disclosure (yes/no) as a predictor of response (Supplementary Table 1). Participants were allocated a ‘yes’ response for the disclosure variable if they had responded ‘yes’ to having disclosed to one or more category of health professional (i.e. one or more of midwife, health visitor or doctor). All those who responded to all three questions concerning disclosure (i.e. whether or not they has disclosed to a midwife, health visitor or doctor) with either a ‘no’ or ‘not applicable’ response were allocated a ‘no’ response for the disclosure variable. All analyses included the following covariates: mothers’ age at giving birth, time passed since giving birth (age in days of their youngest biological child), the number of live births the participant had experienced, country of residence, income, current partner status and the presence of one or more psychiatric conditions (yes or no). A model including disclosure as a predictor was not a better fit than the model without disclosure, *X*^2^(13)=14.84, p=0.42.

Supplementary Table 1 Postnatal appointments

|  | Disclosed | Did not disclose | aOR (95% CI) | p-value | p-value (FDR adjusted) | | | |
| --- | --- | --- | --- | --- | --- | --- | --- | --- |
| I have found it stressful to have health professionals visit my home^a^ |  |  | 1.37 (0.47-4.03) | 0.56 |  | | | |
| N | 28 | 175 |  |  |  | | | |
| Agree | 18 (64%) | 109 (62%) |  |  |  | | | |
| Disagree | 10 (36%) | 64 (37%) |  |  |  | | | |
| Don’t know | 0 (0%) | 2 (1%) |  |  |  | | | |
| Not applicable | 0 (0%) | 0 (0%) |  |  |  | | | |
| I have seen the same professional at each postnatal appointment |  |  | 1.06 (0.45-2.51) | 0.90 |  | | | |
| N | 48 | 298 |  |  |  | | | |
| Yes | 19 (40%) | 115 (39%) |  |  |  | | | |
| No | 29 (60%) | 183 (61%) |  |  |  | | | |
| It is very important to me to see the same health professional at each postnatal appointment^a^ |  |  | 1.24 (0.30-5.18) | 0.77 |  | | | |
| N | 48 | 300 |  |  |  | | | |
| Agree | 44 (92%) | 266 (89%) |  |  |  | | | |
| Disagree | 3 (6%) | 22 (7%) |  |  |  | | | |
| Don’t know | 1 (2%) | 9 (3%) |  |  |  | | | |
| Not applicable | 0 (0%) | 3 (1%) |  |  |  | | | |
| I found it stressful when the health professional I saw was not the person I was expecting to see^a^ |  |  | 2.29 (0.43-12.50) | 0.33 |  | | | |
| N | 47 | 300 |  |  |  | | | |
| Agree | 29 (62%) | 176 (59%) |  |  |  | | | |
| Disagree | 2 (4%) | 31 (10%) |  |  |  | | | |
| Don’t know | 2 (4%) | 9 (3%) |  |  |  | | | |
| Not applicable | 14 (30%) | 84 (28%) |  |  |  | | | |
| I feel that professionals have taken seriously any questions or concerns I have had |  |  | 1.12 (0.45-2.78) | 0.81 | |  | | |
| N | 47 | 295 |  |  | |  | | |
| Agree | 28 (60%) | 173 (59%) |  |  | |  | | |
| Disagree | 16 (34%) | 106 (36%) |  |  | |  | | |
| Don’t know | 0 (0%) | 7 (2%) |  |  | |  | | |
| Not applicable | 3 (6%) | 9 (3%) |  |  | |  | | |
| I felt comfortable asking questions to professionals |  |  | 1.27 (0.52-3.08) | 0.60 | |  | | |
| N | 47 | 293 |  |  | |  | | |
| Agree | 29 (62%) | 169 (58%) |  |  | |  | | |
| Disagree | 18 (38%) | 115 (39%) |  |  | |  | | |
| Don’t know | 0 (0%) | 5 (2%) |  |  | |  | | |
| Not applicable | 0 (0%) | 4 (1%) |  |  | |  | | |
| Professionals have treated me respectfully |  |  | 1.36 (0.52-3.55) | 0.53 | |  | | |
| N | 47 | 295 |  |  | |  | | |
| Agree | 35 (74%) | 208 (71%) |  |  | |  | | |
| Disagree | 12 (26%) | 78 (26%) |  |  | |  | | |
| Don’t know | 0 (0%) | 6 (2%) |  |  | |  | | |
| Not applicable | 0 (0%) | 3 (1%) |  |  | |  | | |
| I have felt negatively judged by professionals^a^ |  |  | 0.96 (0.40-2.33) | 0.94 | |  | | |
| N | 47 | 294 |  |  | |  | | |
| Agree | 23 (49%) | 144 (49%) |  |  | |  | | |
| Disagree | 21 (45%) | 130 (44%) |  |  | |  | | |
| Don’t know | 2 (4%) | 9 (3%) |  |  | |  | | |
| Not applicable | 1 (2%) | 11 (4%) |  |  | |  | | |
| I have felt able to trust professionals |  |  | 1.41 (0.58-3.40) | 0.45 | |  | | |
| N | 47 | 294 |  |  | |  | | |
| Agree | 29 (62%) | 164 (56%) |  |  | |  | | |
| Disagree | 18 (38%) | 122 (42%) |  |  | |  | | |
| Don’t know | 0 (0%) | 4 (1%) |  |  | |  | | |
| Not applicable | 0 (0%) | 4 (1%) |  |  | |  | | |
| I have received enough information about my mental health |  |  | 2.97 (1.21-7.31) | **0.02** |  | | | |
| N | 48 | 299 |  |  |  | | | |
| Agree | 25 (52%) | 100 (33%) |  |  |  | | | |
| Disagree | 19 (40%) | 165 (55%) |  |  |  | | | |
| Don’t know | 2 (4%) | 11 (4%) |  |  |  | | | |
| Not applicable | 2 (4%) | 23 (8%) |  |  |  | | | |
| I have received enough information about looking after my baby |  |  | 2.48 (0.99-6.17) | 0.06 |  | | | |
| N | 48 | 298 |  |  |  | | | |
| Agree | 21 (44%) | 83 (28%) |  |  |  | | | |
| Disagree | 21 (44%) | 172 (58%) |  |  |  | | | |
| Don’t know | 2 (4%) | 13 (4%) |  |  |  | | | |
| Not applicable | 4 (8%) | 30 (10%) |  |  |  | | | |
| I have received enough information about interpreting my baby’s cries |  |  | 1.19 (0.46-3.09) | 0.72 |  | | | |
| N | 48 | 297 |  |  |  | | | |
| Agree | 28 (58%) | 175 (59%) |  |  |  | | | |
| Disagree | 14 (29%) | 93 (31%) |  |  |  | | | |
| Don’t know | 1 (2%) | 5 (2%) |  |  |  | | | |
| Not applicable | 5 (10%) | 24 (8%) |  |  |  | | | |
| I have received enough information about how to play with my baby |  |  | 3.05 (1.22-7.65) | **0.02** |  | | | |
| N | 48 | 299 |  |  |  | | | |
| Agree | 24 (50%) | 95 (32%) |  |  |  | | | |
| Disagree | 18 (38%) | 161 (54%) |  |  |  | | | |
| Don’t know | 1 (2%) | 6 (2%) |  |  |  | | | |
| Not applicable | 5 (10%) | 37 (12%) |  |  |  | | | |
| I am satisfied with the way in which information was presented to me |  |  | 1.55 (0.60-3.96) | 0.36 |  | | | |
| N | 48 | 300 |  |  |  | | | |
| Agree | 30 (63%) | 173 (58%) |  |  |  | | | |
| Disagree | 14 (29%) | 106 (35%) |  |  |  | | | |
| Don’t know | 1 (2%) | 11 (4%) |  |  |  | | | |
| Not applicable | 3 (6%) | 10 (3%) |  |  |  | | | |
| I have had someone to advocate for me during postnatal appointments^b^ |  |  | 2.09 (1.03-4.37) | **0.04** | 0.17 | | | |
| N | 48 | 298 |  |  |  | | | |
| Yes | 32 (67%) | 124 (42%) |  |  |  | | | |
| No | 16 (33%) | 174 (58%) |  |  |  | | | |
| I have found it helpful to have someone to advocate for me during postnatal appointments^b^ |  |  | 1.19 (0.20-10.22) | 0.86 | 0.86 | | | |
| N | 32 | 123 |  |  |  | | | |
| Agree | 28 (88%) | 104 (85%) |  |  |  | | | |
| Disagree | 2 (6%) | 9 (7%) |  |  |  | | | |
| Don’t know | 2 (6%) | 5 (4%) |  |  |  | | | |
| Not applicable | 0 (0%) | 5 (4%) |  |  |  | | | |
| I would have found it helpful to have someone to advocate for me during postnatal appointments^b^ |  |  | 0.25 (0.07-1.51) | 0.14 | 0.28 | | | |
| N | 15 | 173 |  |  |  | | | |
| Agree | 8 (53%) | 99 (57%) |  |  |  | | | |
| Disagree | 5 (33%) | 22 (13%) |  |  |  | | | |
| Don’t know | 0 (0%) | 29 (17%) |  |  |  | | | |
| Not applicable | 2 (13%) | 23 (13%) |  |  |  | | | |
| Satisfaction with midwife appointments |  |  | 0.45 (0.15-1.30) | 0.14 | | |  |  |
| N | 46 | 295 |  |  | | |  |  |
| Satisfied | 21 (46%) | 183 (62%) |  |  | | |  |  |
| Dissatisfied | 11 (24%) | 43 (15%) |  |  | | |  |  |
| Don’t know | 1 (2%) | 6 (2%) |  |  | | |  |  |
| Not applicable | 13 (28%) | 63 (21%) |  |  | | |  |  |
| Satisfaction with health visitor appointments |  |  | 0.92 (0.33-2.54) | 0.88 | | |  |  |
| N | 46 | 295 |  |  | | |  |  |
| Satisfied | 21 (46%) | 152 (52%) |  |  | | |  |  |
| Dissatisfied | 13 (28%) | 75 (25%) |  |  | | |  |  |
| Don’t know | 2 (4%) | 7 (2%) |  |  | | |  |  |
| Not applicable | 10 (22%) | 61 (21%) |  |  | | |  |  |
| Satisfaction with doctor/GP appointments |  |  | 0.65 (0.26-1.64) | 0.36 | | |  |  |
| N | 47 | 296 |  |  | | |  |  |
| Satisfied | 24 (51%) | 188 (64%) |  |  | | |  |  |
| Dissatisfied | 17 (36%) | 83 (28%) |  |  | | |  |  |
| Don’t know | 2 (4%) | 8 (2%) |  |  | | |  |  |
| Not applicable | 4 (9%) | 17 (6%) |  |  | | |  |  |

*Note.* Multivariate binary logistic regression performed

^a^Item reverse scored prior to multivariate analysis. Inverse of aOR and CIs presented

^b^Item not included within multivariate analysis due to survey logic
